# Profiling Detection and Classification of Lameness Methods in British Dairy Cattle Research: A Systematic Review and Meta-Analysis

**DOI:** 10.3389/fvets.2020.00542

**Published:** 2020-08-20

**Authors:** João Sucena Afonso, Mieghan Bruce, Patrick Keating, Didier Raboisson, Helen Clough, George Oikonomou, Jonathan Rushton

**Affiliations:** ^1^Department of Livestock and One Health, Institute of Infection, Veterinary & Ecological Sciences, University of Liverpool, Liverpool, United Kingdom; ^2^School of Veterinary Medicine, College of Science, Health, Engineering and Education, Murdoch University, Murdoch, WA, Australia; ^3^Médecins Sans Frontières – OCA, Manson Unit, London, United Kingdom; ^4^IHAP, Université de Toulouse, INRA, ENVT, Toulouse, France

**Keywords:** lameness, locomotion, meta-analysis, classification, dairy, cattle, British

## Abstract

Lameness is a serious concern in the dairy sector, reflecting its high incidence and impact on animal welfare and productivity. Research has provided figures on its frequency using different methodologies, making it difficult to compare results and hindering farm-level decision-making. The study's objectives were to determine the frequency levels of lameness in British dairy cattle through a meta-analysis approach, and to understand the chronological patterns of how lameness cases are detected and classified in scientific research. A systematic review was conducted using PRISMA-P guidelines for article selection. Random-effects models estimated the pooled frequency measure of lameness with heterogeneity managed through subgroup analysis and meta-regression. Sixty-eight papers were identified, 50 included prevalence and 36 incidence data. The pooled prevalence of lameness in British dairy cattle was estimated at 29.5% (95% CI 26.7–32.4%) whilst all-cause lameness incidence rate indicated 30.9 cases of lameness per 100 cow-years (95% CI 24.5–37.9). The pooled cause-specific lameness incidence rate per 100 cow-years was 66.1 (95% CI 24.1–128.8) for white line disease, 53.2 (95% CI 20.5–101.2) for sole ulcer, 53.6 (95% CI 19.2–105.34) for digital dermatitis, with 51.9 (95% CI 9.3–129.2) attributable to other lameness-related lesions. Heterogeneity levels remained high. Sixty-nine papers contributed to a chronological overview of lameness data source. Although the AHDB Dairy mobility scoring system (MSS) was launched in the UK in 2008 and adopted shortly after by the British Dairy sector as the standard tool for assessing lameness, other methods are used depending on the investigator. Automated lameness detection systems may offer a solution for the subjective nature of MSSs, yet it was utilized in one study only. Despite the recognition of under-reporting of lameness from farm records 22 (31.9%) studies used this data source. The diversity of lameness data collection methods and sources was a key finding. It limits the understanding of lameness burden and the refinement of policy making for lameness. Standardizing case definition and research methods would improve knowledge of and ability to manage lameness. Regardless of the measurement method lameness in British dairy cattle is high.

## Introduction

Livestock production has changed quite substantially over the last century, in response to supply issues with regards to technological development, and socio-demographic changes with increasing numbers of people and their wealth leading to greater demand for animal products. Extensive subsistence systems have given way to intensive commercial structures (1, 2), which has led to a change in the production environment and consequently to the upsurge and/or increase of the incidence of production diseases, resulting in reduced animal welfare (3–5). The increasing global consciousness regarding animal and food production, and the scientific body of evidence pressures political leaders to debate and legislate animal production toward more environmentally sustainable and higher-welfare standard systems (6).

Lameness is currently one of the main health concerns facing the livestock sector, particularly in the dairy cattle industry. According to the Agriculture and Horticulture Development Board (AHDB) Dairy (formerly known as DairyCo), a UK non-departmental public body funded by farmers and organizations in the food supply chain, it encompasses any foot or leg condition of infectious or non-infectious (environmental and/or farm management factors) etiology leading to abnormal locomotion (7). It has serious implications in terms of animal welfare (8–12) and significant impact in production as a result of reduced milk yield, reproductive performance and weight gain, and increased involuntary culling (13–17). Adding expenditure for the treatment of affected animals to production losses, Willshire and Bell (18) estimated that clinical lameness costs the typical UK dairy herd (defined as 112 Holstein-Friesian cows fed a partial mixed ration, with an average yearly milk yield of 6,885 liters/cow and an average calving index of 410 days) £7,499.30 per year, which translates into 0.97 pence per liter. Previously, Kossaibati and Esslemont (19) estimated the costs of lameness in a British 100-cow herd at £1,715 per year. Bennett and IJpelaar (20) estimated the costs of endemic livestock diseases in the UK, while also providing a score of the welfare impact of those conditions in the animal population. In this exercise lameness cost the UK cattle livestock sector 53.5 million sterling pounds—second to mastitis, the most costly disease—and ranked first in the welfare impact evaluation (20). Additionally the impact of lameness in a cow's mobility and behavior can discourage the adoption of technologies developed for improving the business efficiency such as the Automatic Milking Systems (AMS), which rely on the voluntary attendance of the cow to the milking robot (21, 22). Moreover, being associated with increased lying behavior it is probable that lameness augments the risk of mastitis—the most costly aliment in dairy cattle among production diseases (23).

There is, however inconsistent data on lameness, be it in terms of availability or accessibility (24). This is particularly important when estimating or calculating the frequency of disease; a key parameter for animal health economic analyses. The reliability of the estimates is closely associated with the quality of data available. Farm records are commonly used as source of data for calculating disease frequency, yet studies consistently conclude that lameness in cows is under-reported by farmers (25–27). Whay et al. (28) reported that farmers would underestimate lameness prevalence by 17% when compared with the observations from an independent and trained assessor. Leach et al. (29) reported this difference to be close to 30%, with the mean farmer-reported lameness prevalence at around 7%. Scoring systems with ordinal scales based on animal's posture and walking pattern were developed to aid the detection of lameness. However, the subjectivity inherent in assigning scores and the diversity of scoring instruments used contributes to inconsistencies (30–33). The lack of a standard definition for lameness predisposes misclassification errors (26, 34–36); and the diversity of study designs, and data collection and analysis methodology used in research hampers our ability to compare results across different studies, making it difficult for people involved in the milk value chain to make informed decisions (2, 37). Without a standard method of assessment lameness frequency levels it is hard to understand the trends of the health condition through time and its burden, and to assess the effectiveness of the measures for managing it.

The objectives of this study were to:

- Conduct a meta-analysis to estimate the pooled prevalence and incidence rate of lameness in British dairy cattle since 1823.
- Chronologically analyze the use of different lameness detection and classification methods used in British dairy cattle lameness research, to investigate temporal trends and determine whether specific methods have been used consistently.

## Materials and Methods

A systematic review to identify papers reporting frequency of lameness in British dairy cattle was conducted in six electronic scientific literature databases—*Agricola, Cab Direct, Cochrane Library, PubMed, Scopus*, and *Web of Science* (all databases) on the 4th of January 2020. The systematic review protocol was developed based on the Cochrane guidelines (38), and the PRISMA-P (Preferred Reporting Items for Systematic reviews and Meta-Analyses) statement (39), with specific modifications for a systematic review reporting measures of disease frequency, as recommended by the Joanna Briggs Institute (40). The search was limited to peer reviewed articles, published since 1823 in English. The population search terms were (dairy AND cattle) AND (UK OR Britain OR British OR kingdom). The outcome search terms were (lameness AND (prevalence OR incidence). The following code was used for all six databases considered: (dairy AND (cattle OR cow^*^) AND (UK OR British OR Britain OR kingdom) AND (lame^*^ OR locomotion) AND (incidence OR prevalence). The search through *Scopus* was limited to abstract, title and keywords. A synthesis of the diagnostic protocols used was also conducted, with the objective of identifying temporal patterns in the use of different methodologies and to determine if any diagnostic protocol has been used consistently over time.

EndNote X9 (Thompson Reuters) bibliographic software was used to manage citations. Duplicate entries were identified, using the automatic function in EndNote and manually during the screening process, by considering the author, the year of publication, the article title, and the volume, issue, and page numbers of the source. In questionable cases, the abstracts or full texts were compared. Conference papers reporting studies that were subsequently published in journals were considered duplicates.

### Eligibility Criteria

**Figure 1:**
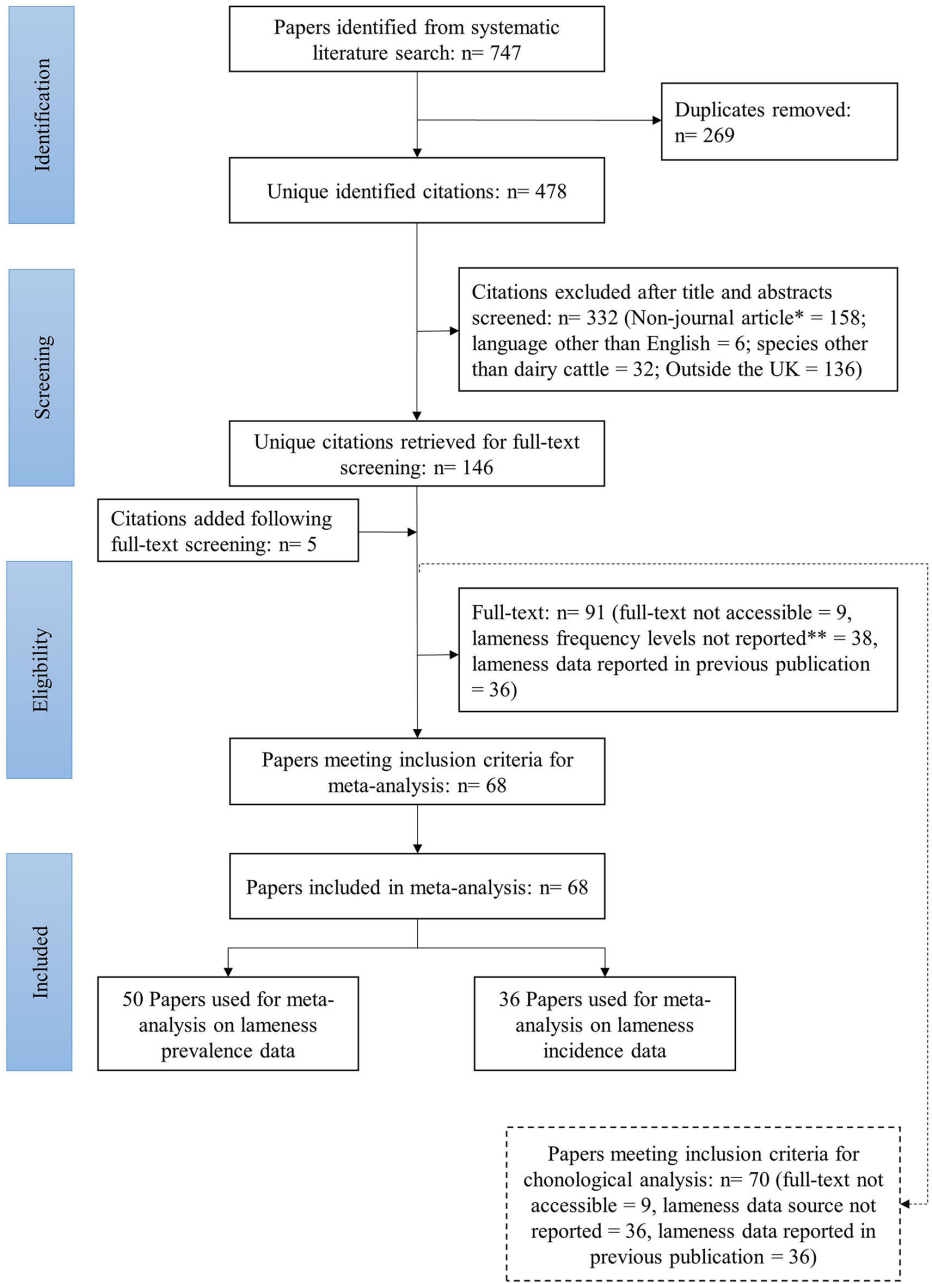
Flow diagram of studies identified by the systematic review and their selection process and inclusion for the meta-analysis on lameness frequency levels in British dairy cattle (*short communications, letters, self-assessments, and review articles were excluded, **if lameness frequency levels were reported but no information on population at risk/denominator was provided/retrievable the paper was excluded).

The systematic review and article selection for the meta-analysis followed the PRISMA guidelines (39) according to the diagram in Figure 1.

Titles and abstracts from the records identified in the search were screened for eligibility based on the population, intervention or exposure, comparator group, outcome, study design (PICOS) approach using the following criteria: (i) Population: British dairy cattle; (ii) Outcome: lameness prevalence and/or incidence, lameness causing foot lesions; and (iii) Study design: Randomized controlled trials, cohort studies, case-control studies, cross-sectional studies, case reports and outbreak investigations were all eligible for inclusion if they reported number of dairy cows that were lame (numerator) and the study population (denominator), or if the same could be calculated. Only studies published in peer-reviewed journals were included, with no date restriction. Language of publication was restricted to English. Papers that reported data from previous publications were excluded as to have only one entry per data collection exercise.

If the study met all the inclusion criteria but didn't provide data on the number of lame cows and/or study population the corresponding author was contacted via email in an effort to retrieve the missing information and for clarification. If the corresponding author was not available, one of the co-authors was contacted. If the author(s) did not reply or could not provide the information requested the paper was excluded from the meta-analysis.

In addition to the references identified through the systematic review, five other papers were identified (41–45) following a backward search (also known as chain search) on the papers admitted for full-text screening, and added to the database (Figure 1). The backward search involves identifying references cited in an article that may be relevant for the study in question (46).

For the chronological analysis of the use of different lameness detection and classification methods, all studies that underwent full-text screening were considered. Papers with information on the lameness data source were included, regardless of them meeting inclusion criteria regarding lameness data availability. Papers that reported the same original research were considered duplicates and were removed (Figure 1). The usage of lameness detection and classification methods through time was analyzed by means of a histogram.

### Data Extraction

Data extraction was performed by one reviewer (JSA) and checked for accuracy by MB. Any ambiguities were discussed and consensus reached. The data extracted from the records were based on the recommendations of PRISMA-P (47) and included: (i) study characteristics (authors, year of publication, year or years of data collection, study type—experimental or observational, study design, sample size, sampling strategy); (ii) population data (breed, production system, milking system, grazing regime, housing system, study unit); (iii) outcome data (lameness classification method, lameness assessment frequency, lameness assessment observer, measure of disease frequency); and (iv) numerator and denominator data (number of lame cows, total number of cows in the study population, number of lameness events, population at risk, study duration). The PRISMA-P checklist can be consulted in the Supplementary Material section.

### Database Management and Parameters

Microsoft Excel (48) was used to create a database with the data extracted from the papers. Most variables contained a substantial number of categories, or range in values. This would result in a high number of strata with small number of papers when conducting the analysis, at the expense of statistical power. In order to solve this problem new binary variables were created based on the values extracted from the papers (Supplementary Table 2). Papers reported lameness for different study units depending on their study population: heifers, cows, lactations and culled cows. Papers reporting lameness per lactation were included in the category “cow,” assuming that each lactation represents a dairy cow. Sample size was used to create a new binary variable, and to explore the potential effect of smaller sample sizes on lameness estimates. The cut-off for animal sample size was based on the median of the animal sample size for the identified studies. The median was 1,237, the cut-off was defined at 1,230. The choice of the farm number was based on the median of the farm sample size for the identified studies. The median was 4, and the farm sample size cut-off was defined as 5. Given the increasing awareness to the lameness issue through time the years of the start of data collection was used to create five different variables. Different cut-offs were defined: 1995, 2000, 2005, 2008, and 2010. The last 2 years reflect the adoption of the AHDB Dairy mobility scoring system as the dairy industry standard and the implementation of the AHDB Dairy Healthy Feet program, respectively (49). The variables were named *Start of data collection (year)* as to indicate that the cut-off refers to the year data collection was initiated. The five variables were numbered from 1 to 5 with respect to the chronology of the cut-off: 1 would stand for the year 1995 as the cut-off and 5 for the year 2010.

As the incidence rate of lameness was reported in different time units, the incidence data were extracted and standardized for 100 animal-years. To explore the underlying causes of lameness the following grouping of lesions was defined, based on Griffiths et al. (50):

- White Line Diseases (WLD) and Abscess → White Line Disease.
- Sole Ulcer and Sole Hemorrhage/ Bruising → Sole Lesions.
- Bovine Digital Dermatitis and Interdigital Phlegmon/Foul-in-the-foot/Footrot → Infectious-nature lameness.
- All other lesions → Other.

The identified papers reported lameness based on three distinct study units—cow, heifer and culled cow. Due to the inherent differences of dairy cows in various life stages, and to the fact that reporting disease frequency according to culling reason does not necessarily reflect the herd's disease incidence or prevalence, a pooled-estimate was not considered to be appropriate. Therefore, papers were grouped according to the moment of the production cycle at which lameness frequency was reported, and a meta-analysis conducted on these sub-sets of data.

### Methodological Quality Assessment (Risk of Bias)

As advocated by the Cochrane the quality assessment of the studies included in the meta-analysis was focused on the methodological aspects, hence risk of bias (51). The lameness frequency levels reported in the papers included for the meta-analysis were assessed as to their potential risk of bias. This exercise followed the QUADAS2 approach (52) and an adapted tool (see Risk Bias Assessment in Supplementary Material) was used to evaluate the potential risk of bias of a set of components and its applicability. The tool was piloted by two researchers (JSA and an invited researcher—BG—who was not otherwise involved in the study) on two randomly selected papers. If there was no agreement between the two researchers when assessing the papers, the tool was revised and re-piloted on two other randomly selected papers. A paper was considered to have a low overall risk of bias if the risk of bias and applicability concerns were low.

### Analysis

The primary outcome measure was incidence rate or prevalence of lameness in British dairy cattle. Analysis was conducted using RStudio statistical software (version−1.2.1335; R Foundation for Statistical Computing, Vienna, Austria) using the *meta* and *metafor* packages (53). The *metaprop* function was used to conduct the meta-analysis on the prevalence data and the *metarate* function for the incidence data.

The fixed-effect model disregards the between-study variance and assumes that the methods and underlying population from which the sample was drawn are equal between the different studies. These assumptions did not seem to fit well given the heterogeneity of the methods and sample population between the identified studies. For that reason a random effects model was chosen over a fixed-effect model (54, 55). Data were assessed for skewedness. As it was not normally distributed, data were transformed using arcsine transformation. A sensitivity analysis was conducted comparing the results obtained using the arcsine transformation with those obtained when using other available data transformation methods (56). The GLM model was only used for the prevalence data.

With the exception of the GLM model, all models used the inverse variance method for pooling the estimate of the lameness frequency level. Confidence intervals for individual studies were estimated through the normal approximation interval based on the summary measure (pooled lameness prevalence for studies reporting lameness prevalence and pooled lameness incidence for studies reporting lameness incidence). The DerSimonian-Laird (DL) estimate was used to calculate the between-study variance τ^2^ in all models but the GLMM (57, 58). In the latter the Maximum-likelihood estimator was used (59).

#### Heterogeneity on the Reported Lameness Frequency Levels Between Studies

In the realm of a meta-analysis heterogeneity is defined as the variability of the measure of interest across the selected studies, which can arise from different reasons such as different study methodologies or sampling strategies. Understanding and quantifying heterogeneity is important to allow the researcher to appreciate the range of values the summary measure can take (51). A high heterogeneity level indicates that the variability of the values reported across the individual studies is very large. Studies reporting extreme values that deviate substantially from the summary measure can increase heterogeneity. There could also be a factor or factors, also referred to as moderator(s), by which studies can be grouped that can justify the high levels of heterogeneity (e.g., study design, study type, gender, age of study population). As it may not be adequate to provide a summary measure when heterogeneity levels are high, methods are applied to reduce it (60). A two-step approach was used to address heterogeneity. The first-step was to identify outliers and influential studies. The forest plot was assessed and studies whose 95% confidence intervals did not overlap with that from the pooled estimate were identified. A set of tests followed to formally assess the influence of the outlying effect of individual studies on the pooled estimate by means of the function *influence*. Papers that had a strong influence on the overall estimate were removed from the meta-analysis. The second step was to use a moderator analysis, first by sub-group analysis (univariate), grouping the studies by factors that could explain the heterogeneity, followed by a multiple meta-regression if more than one factor was identified as a predictor of the variance between studies (55). Factors providing a *P*-value of 0.1 or below in the test for moderators were considered moderators and added to the multiple meta-regression model. The model for the multiple meta-regression was developed using the *glmulti* package, and according to the multimodel inference method in which all possible combinations of the identified predictors are explored and parsimony is rewarded (60). The second step was only conducted if there were at least 10 papers, and if there were at least 5 papers per subgroup (60).

Risk factors for lameness were explored in the moderator analysis (breed, grazing regime, calving pattern, housing system, and milking system). Additionally, factors that could have an influence on the reported levels of lameness were also considered in the moderator analysis: lameness data source (records vs. MSS and/or ALDS), study type, study design, study farm(s) location, year of start of data collection and sample size (55).

## Results

### Chronological Overview of Lameness Classification Methods

Out of the 151 papers that were considered for full-text screening, 70 papers were eligible for the chronological analysis. One paper (61) had been published sometime in the past when compared with the other studies and for this reason was excluded from the analysis.

**Figure 2:**
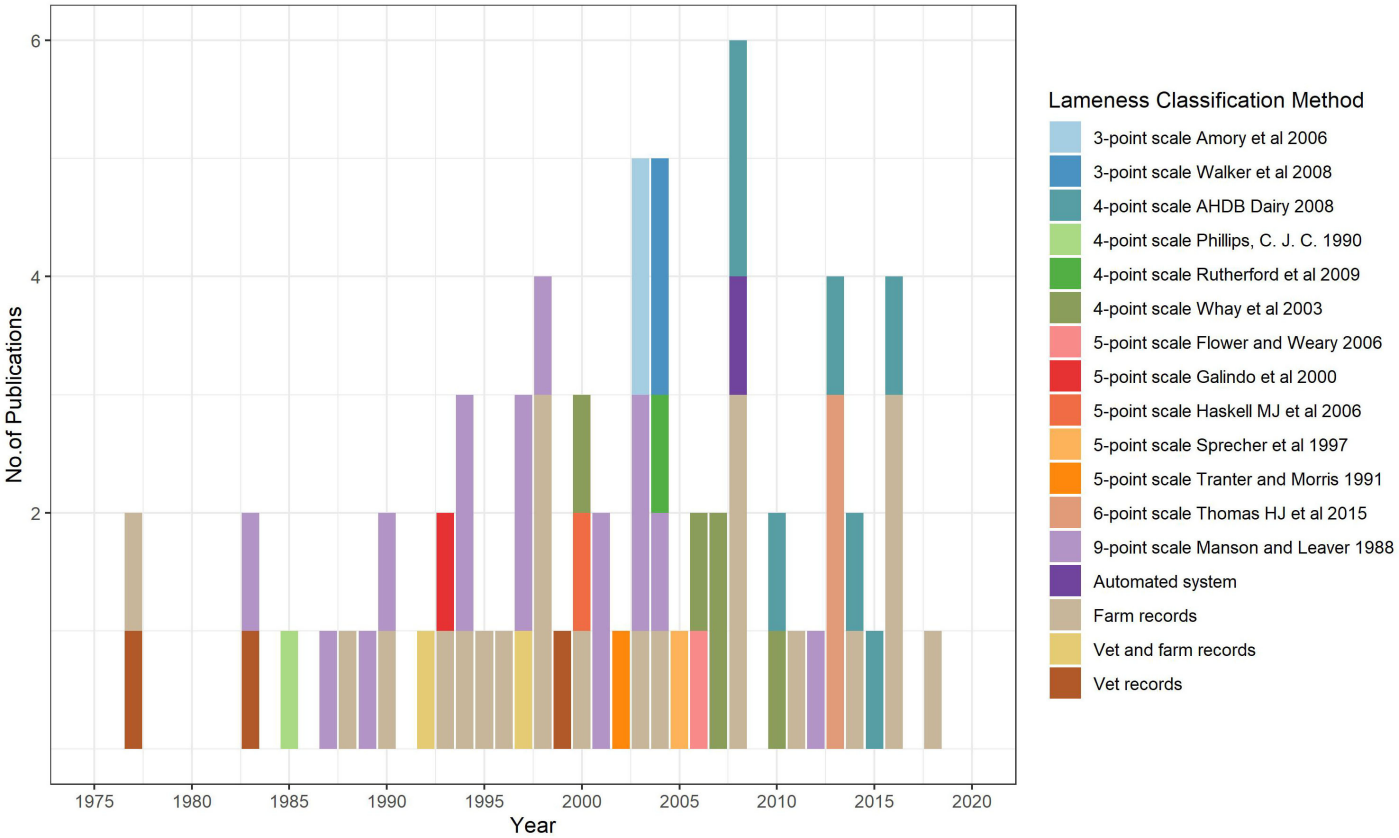
The number of publications in each year, according to the start of the data collection period, and specific lameness detection and classification methods used in research in British dairy cattle since 1975 (each bar represents a paper and the color the method used).

Overall 17 different lameness data sources were identified, among records and mobility scoring systems (MSS). Up until the year 2000 only 3 distinct MSS had been used in lameness research in British dairy cattle. From 2000 onwards another 10 MSS were used in publication regarding the study of lameness in the same population (Figure 2). Just over 20% of all the papers used the 9-point scale Manson and Leaver 1988 to collect lameness data, being the most commonly used MSS. The 4-point scale AHDB Dairy 2008 ranked second with about 10% of the papers making use of this MSS. Despite the existence of different mobility scoring tools for assessing lameness in dairy cattle, farm records are still a commonly used lameness data source by research (Table 1). Five out of the eight papers making use of data that started being collected from 2014 onwards sourced their lameness data from farm records. Only one paper made use of automated lameness detection systems (ALDS) for collecting data (Figure 2).

**Table 1:** Relative distribution of lameness data sources across the identified papers (*n* = 69) for the chronological overview of lameness classification methods.

**Lameness data source**	**% (*n*)**
Farm records	31.9% (22)
9-point scale Manson and Leaver (1988) (62)	21.7% (15)
4-point scale AHDB Dairy (2020) (7)	10.1% (7)
4-point scale Whay et al. (2003) (28)	7.2% (5)
6-point scale Thomas et al. (2016) (63)	4.3% (3)
3-point scale Amory et al. (2006) (31)	4.3% (3)
3-point scale Walker et al. (2008) (64)	2.9% (2)
Vet and farm records	2.9% (2)
Vet records	2.9% (2)
4-point scale Phillips (1990) (65)	1.4% (1)
4-point scale Rutherford et al. (2009) (66)	1.4% (1)
5-point scale Flower and Weary (2006) (67)	1.4% (1)
5-point scale Galindo and Broom (2000) (68)	1.4% (1)
5-point scale Haskell et al. (2006) (69)	1.4% (1)
5-point scale Sprecher et al. (1997) (70)	1.4% (1)
5-point scale Tranter and Morris (1991) (71)	1.4% (1)
Automated system	1.4% (1)

### Meta-Analysis on Lameness Frequency Levels

Of the 151 potentially eligible studies, 68 were included in the meta-analysis (Figure 1), 16 references were found to have missing data on number of lame animals or population at risk, and author(s) were contacted to see if the information could be provided. Data could not be retrieved on twelve papers—on four papers authors could not be reached, and on eight papers authors weren't able to provide the data. Papers were published from 1946 until 2019, with 75% based on data collected from 1995 onwards (Table 2). Fifty had lameness prevalence data whereas 36 had incidence data (Figure 1).

**Table 2:** Summary statistics of the final set of studies reporting lameness frequency levels in British dairy cattle (*n* = 68).

**Variable**	**Category**	**% of papers (*n*)**
Start of data collection (year) 1	Before 1995	23.5% (16)
	1995 and onwards	76.5% (52)
Start of data collection (year) 2	Before 2000	38.2% (26)
	2000 and onwards	61.8% (42)
Start of data collection (year) 3	Before 2005	58.8% (40)
	2005 and onwards	41.2% (28)
Start of data collection (year) 4	Before 2008	66.2% (45)
	2008 and onwards	33.8% (23)
Start of data collection (year) 5	Before 2010	76.5% (52)
	2010 and onwards	23.5% (16)
Breed	Holstein/Friesian/Holstein-Friesian	67.6% (46)
	Other 4	20.6% (14)
	Not reported	11.8% (8)
Calving pattern	Year-round	23.6% (16)
	Other	27.9% (19)
	Not reported	48.5% (33)
Grazing regime	Grazing	35.3% (24)
	Other	30.9% (21)
	Not reported	33.8% (23)
Housing system	Cubicle	48.5% (33)
	Other	22.1% (15)
	Not reported	29.4% (20)
Milking system	Conventional	57.4% (39)
	Other	2.9% (2)
	Not reported	39.7% (27)
Study farm(s) belonging to research institute	Yes	23.5% (16)
	No	76.5% (52)
Study Type	Experimental	26.5% (18)
	Observational	73.5% (50)
Study design	Cross-sectional	10.3% (7)
	Longitudinal	57.4% (39)
	Negatively controlled RCT	2.9% (2)
	Positively controlled RCT	1.5% (1)
	Retrospective longitudinal	27.9% (19)
Study unit	Cow	77.9% (53)
	Culled cow	5.9% (4)
	Heifer	10.3% (7)
	Lactation	5.9% (4)
Lameness data source	Mobility scoring system	57.3% (39)
	Records	41.2% (28)
	Other	1.5% (1)
Sample size a	Less than 1,230 animals	52.9% (36)
	1,230 animals or more	47.1% (32)
Sample size b	Less than 5 farms and/or 1,230 animals	63.2% (43)
	At least 5 farms and 1,230 animals	36.8% (25)

The main breed of animals were Holstein, Friesian, or Holstein-Friesian, with about 70% of all studies described the animals as belonging to these breeds (Table 2). A significant proportion of studies did not report data on calving pattern, grazing regime, housing system, and milking system. A quarter of the studies were conducted in farms that belonged to research institutes. Most studies were observational (73.5%). In terms of study design 57.4% were longitudinal, and roughly 10.3% of them were cross-sectional. The majority of papers (77.9%) reported on cows, regardless of their age. A small number of papers (10.3%) focused their research on heifers. Two out of every five studies relied on records for their lameness data. More than half of all studies based their research on a sample of <5 farms and/or 1,230 animals (65.6%) (Table 2).

Sections Meta-Analysis on Lameness Prevalence Levels and Meta-Analysis on Lameness Incidence Rate Levels will concentrate on the results from the papers reporting lameness prevalence and incidence rate at cow level.

#### Meta-Analysis on Lameness Prevalence Levels

Fifty studies were included in the meta-analysis on lameness prevalence data. Forty-two, five and three studies reported lameness prevalence at cow, heifer and culled cow level, respectively (Supplementary Table 4). The results presented in this section are based on the 42 papers reporting lameness prevalence at cow level. Pooled estimates are provided along with their 95% confidence intervals (CI). The 95% prediction intervals (PI) are also provided except for the sub-group analysis results.

**Figure 3:**
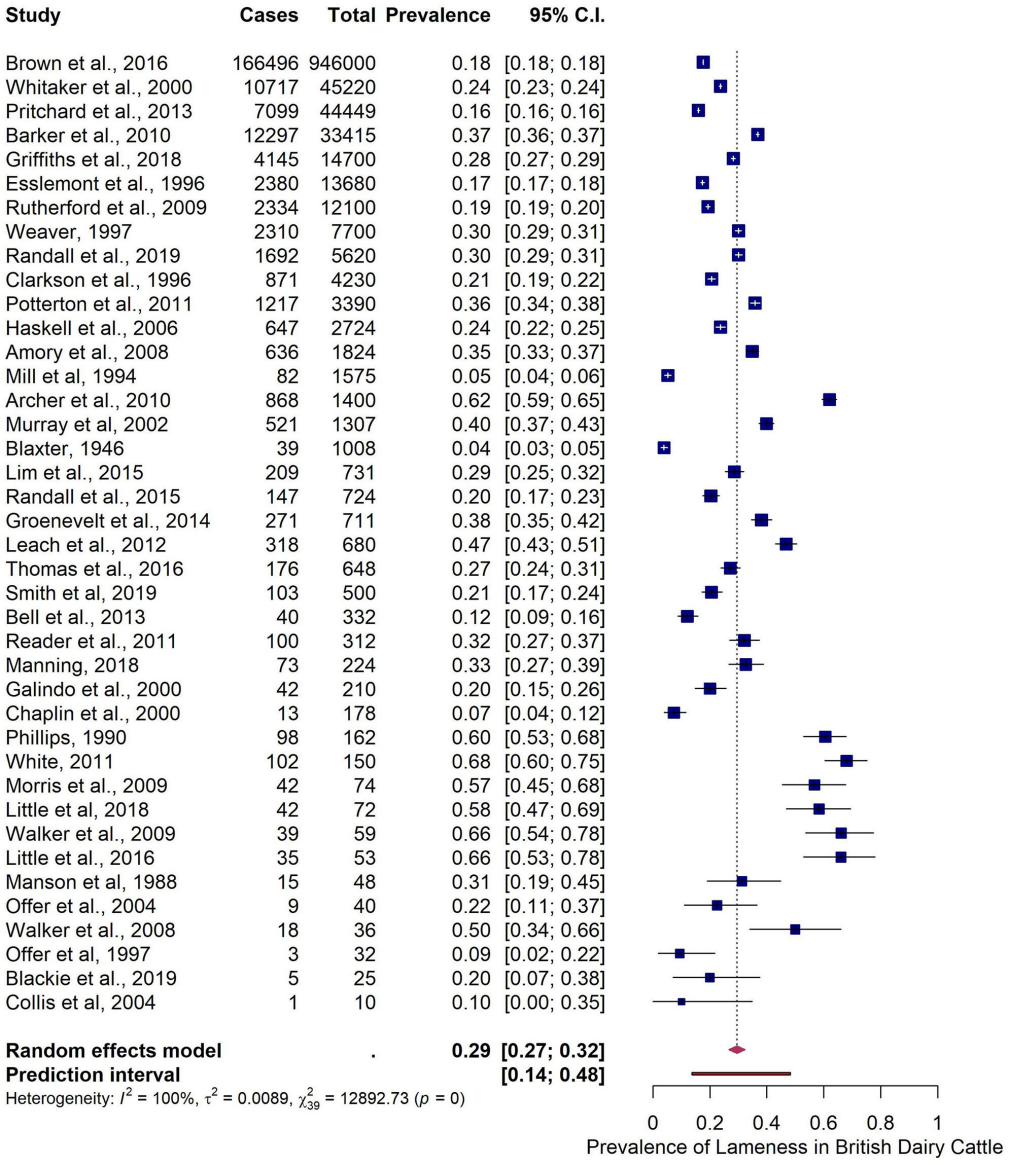
Meta-analysis on reported lameness prevalence in British dairy cattle from identified studies.

Two outliers were identified. The overall pooled estimate for the prevalence in British dairy cattle after the removal of the outliers was 29.5% (95% CI 26.7–32.4% and 95% PI 13.8–48.2%). Heterogeneity was present and extensive (Table 3 and Figure 3).

**Table 3:** Summary of the results from the meta-analysis of studies reporting lameness prevalence at cow level using arcsine data transformation method.

**No of studies**	**Pooled prevalence**	**95% CI**	**95% PI**	**Heterogeneity measures**			
				**Cochran's Q**	***P*-value Q**	**Tau^**2**^**	***I^**2**^*(%)**
**Before outlier identification and removal**							
42	0.299	0.261–0.339	0.087–0.572	34975	<0.001	0.019	99.9
**After outlier identification and removal**							
40	0.295	0.267–0.324	0.138–0.482	12892	<0.001	0.009	99.7

The pooled estimates between subgroups when papers were grouped per variables *Start of data collection (year) 1, Start of data collection (year) 2, Start of data collection (year) 3, Start of data collection (year) 4*, and *Start of data collection (year) 5* were statistically different (*p*-value for the test of moderator <0.1) and therefore were considered as moderators for the meta-regression (Table 4). As there were <5 papers in one of the categories for variable *Milking System* no sub-group analysis was conducted on this factor.

**Table 4:** Sub-group analysis (univariate analysis) for the papers reporting lameness prevalence at cow level.

**Moderator**	**Subgroup**	**No of studies per subgroup**	**Pooled prevalence per subgroup**	**95% CI**	***P*-value for QM**	**Residual heterogeneity (H)**	**Residual heterogeneity (*I^**2**^*) (%)**
Lameness data source	Mobility scoring system	27	0.294	0.253–0.337	0.672	13.04	99.4
	Records	13	0.283	0.251–0.315			
Study type	Observational	29	0.279	0.249–0.311	0.263	18.11	99.7
	Experimental	11	0.338	0.241–0.443			
Study farm(s) location	Not at Research Institute	32	0.289	0.258–0.320	0.667	18.39	99.7
	At Research Institute	8	0.325	0.173–0.499			
Study design	Cross-sectional	7	0.246	0.182–0.316	0.127	13.86	99.5
	Other	33	0.304	0.278–0.329			
Breed	Holstein^a^	26	0.279	0.255–0.305	0.976	13.40	99.4
	Other	9	0.278	0.214–0.348			
Grazing regime	Other	12	0.308	0.268–0.349	0.578	9.2	99.0
	Grazing	16	0.333	0.256–0.415			
Housing system	Multiple	9	0.293	0.233–0.357	0.189	12.56	99.4
	Cubicle	20	0.357	0.285–0.433			
Calving pattern	Other	11	0.371	0.324–0.420	0.697	8.23	98.5
	Year-round	8	0.334	0.171–0.521			
Start of data collection (year) 1	1995 and onwards	33	0.319	0.287–0.353	0.004^*^	18.36	99.7
	Before 1995	7	0.195	0.128–0.273			
Start of data collection (year) 2	2000 and onwards	27	0.349	0.301–0.399	<0.001^*^	18.33	99.7
	Before 2000	13	0.200	0.163–0.240			
Start of data collection (year) 3	2005 and onwards	19	0.368	0.305–0.433	<0.001^*^	18.28	99.7
	Before 2005	21	0.231	0.199–0.263			
Start of data collection (year) 4	2008 and onwards	14	0.368	0.302–0.436	0.005^*^	16.37	99.6
	Before 2008	26	0.258	0.219–0.298			
Start of data collection (year) 5	2010 and onwards	11	0.356	0.297–0.418	0.023^*^	16.02	99.6
	Before 2010	29	0.273	0.234–0.313			
Sample Size a	1,230 animals or more	17	0.263	0.225–0.302	0.169	18.26	99.7
	less than 1,230 animals)	23	0.328	0.245–0.417			
Sample Size b	More than 5 farms and 1,230 animals	14	0.265	0.218–0.314	0.171	15.20	99.6
	less than 5 farms and/or 1,230 animals	26	0.319	0.259–0.380			

a *Herds which cows were mainly Holstein, Friesian and/or Holstein-Friesian*.

* *Variables considered as moderators*.

**Figure 4:**
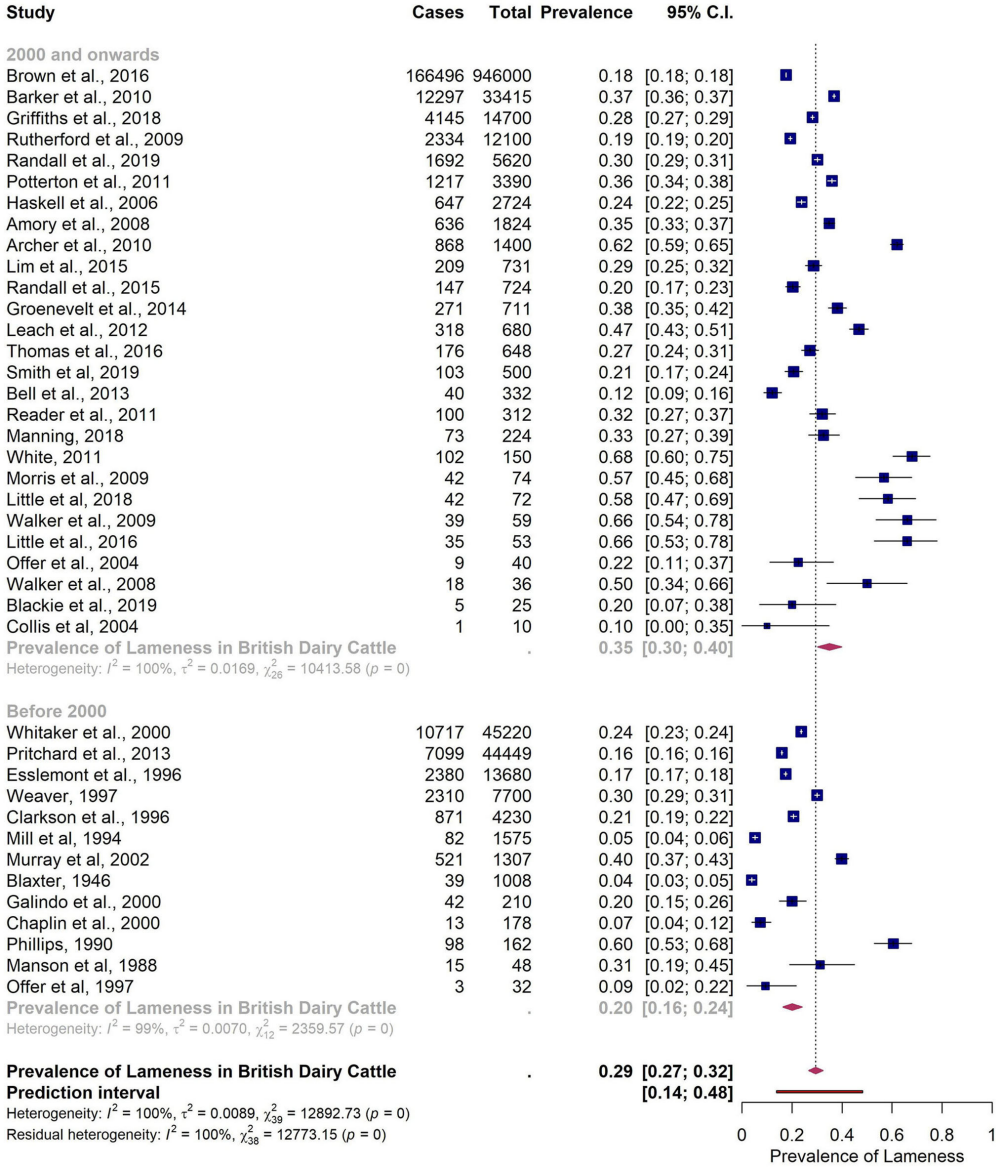
Subgroup analysis of reported prevalence of lameness in British dairy cattle at cow level with Start of data collection (year) 2 as a moderator (year 2000 as cut-off).

The five identified predictors were used in the multiple meta-regression model. The model with the moderator *Start of data collection (year) 2* (year 2000 as cut-off) was the most parsimonious model. The pooled estimate for papers published from 2000 onwards was 34.9% (95% CI 30.1–39.9%), roughly 15% more when compared with the pooled estimate from studies published before the year 2000 (20.0%; 95% CI 16.3–24.0%) (Figure 4).

#### Meta-Analysis on Lameness Incidence Rate Levels

Lameness incidence rate data was extracted from thirty-six studies, thirty-one of which reported the measure at cow level. One paper reported incidence per culled cow whereas the remaining four reported at heifer level (Supplementary Table 5). Additionally, data on the underlying cause of lameness were extracted from papers that reported it and a meta-analysis conducted. The results presented are based on incidence rate data from papers reporting cases at the cow level.

**Figure 5:**
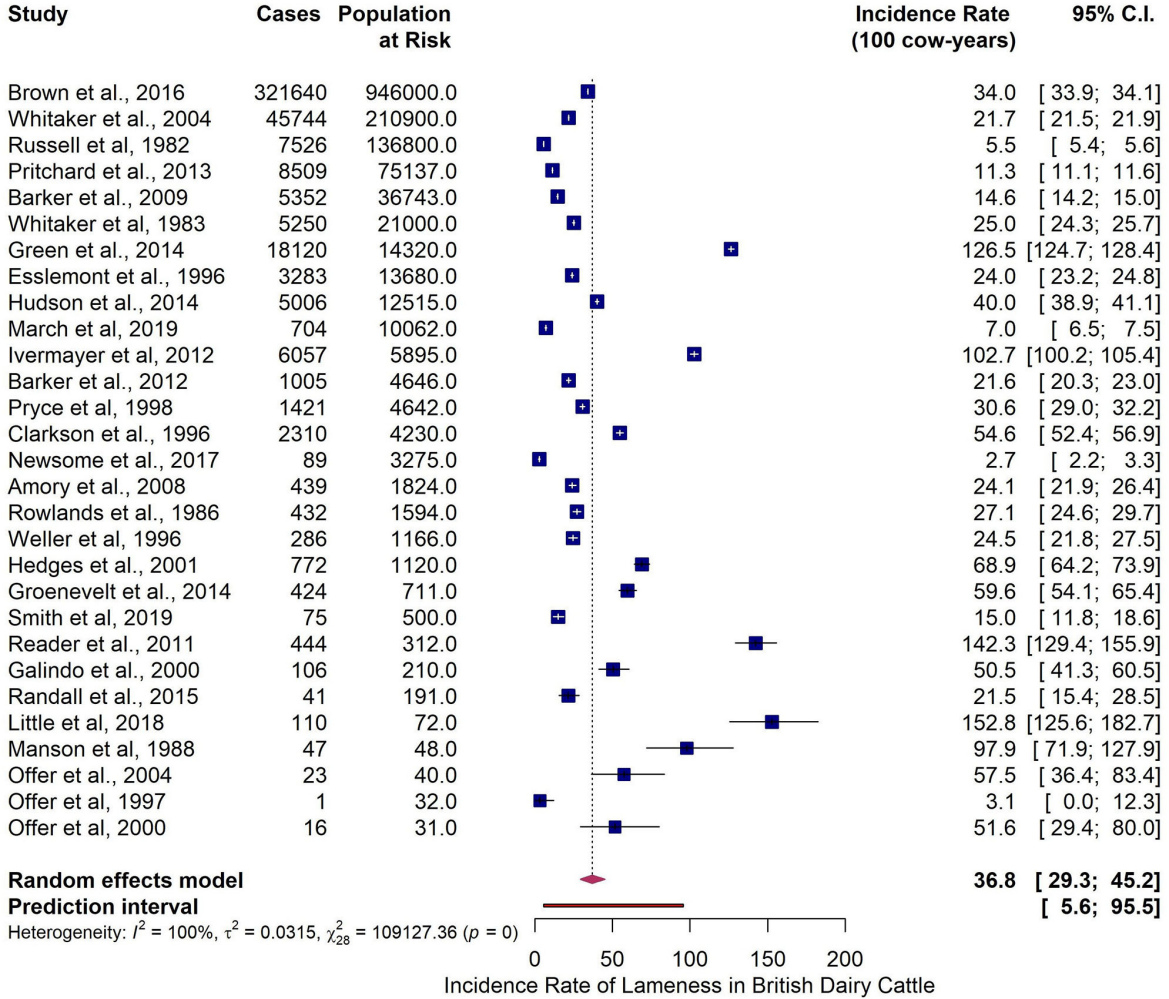
Meta-analysis on reported lameness incidence rate (100 cow-years) in British dairy cattle from identified studies after outlier removal.

Two studies were identified as outliers and removed from the analysis. After the removal of the outliers the overall pooled estimate for all-causes incidence rate in British dairy cattle was 36.8 cases per 100 cow-years (95% CI 29.3–45.3 and 95% PI 5.6–95.5). Heterogeneity was present and extensive (Table 5 and Figure 5).

**Table 5:** Summary of the results from the meta-analysis of studies reporting lameness incidence rate (100 cow-years) at cow level using the arcsine data transformation method, before and after outlier removal.

**No of studies**	**Pooled Incidence rate (100 cow-years)**	**95% CI**	**95% PI**	**Heterogeneity measures**			
				**Cochran's Q**	***P*-value Q**	**Tau^**2**^**	***I^**2**^*(%)**
**Before outlier identification and removal**							
31	45.2	36.9–54.3	8.8–109.7	112985	<0.001	0.033	100.0
**After outlier identification and removal**							
29	36.8	29.3–45.2	5.6–95.5	109127	<0.001	0.032	100.0

Few studies provided information on the lameness underlying cause, ranging from 11 (*Sole Ulcer, Sole Hemorrhage/Bruising* category) to 8 (*White Line Disease, White Line Disease and Abscess, Bovine Digital Dermatitis*, and *Other lesions* categories). The pooled incidence rate per 100 cow-years was 66.1 (95% CI 24.1–128.8), 53.2 (95% CI 20.5–101.2), 53.6 (95% CI 19.2–105.34), and 51.9 (95% CI 9.3–129.2) for *White Line Disease, Sole Ulcer, Bovine Digital Dermatitis*, and *Other lesions*, respectively. As with the meta-analysis on the all-causes incidence rate data, the heterogeneity was present and high for all lameness-related lesions (Table 6).

**Table 6:** Summary of the results from the meta-analysis of studies reporting lameness causing lesions incidence rate (100 cow-years) at cow level using the arcsine data transformation method, after outlier removal.

**Lesion(s)**	**No of studies (No of outliers)**	**Pooled incidence rate (100 cow-years)**	**95% CI**	**95% PI**	**Heterogeneity measures**			
					**Cochran's Q**	***P*-value Q**	**tau 2**	***I*^**2**^ (%)**
Wld^a^	7 (1)	66.1	24.1–128.8	14.4–402.4	23947	<0.001	0.188	100.0
Wldabs^b^	6 (2)	75.2	25.5–151.2	24.0–494.8	23881	<0.001	0.205	100.0
Su^c^	9 (1)	53.2	20.5–101.2	10.5–317.9	26148	<0.001	0.179	100.0
Sushb^d^	10 (1)	46.6	22.2–79.8	1.9–226.3	44594	<0.001	0.115	100.0
Bdd^e^	8 (1)	53.6	19.2–105.3	13.6–335.9	26129	<0.001	0.179	100.0
Bddfr^f^	7 (2)	60.2	26.6–107.5	3.6–303.2	43872	<0.001	0.123	100.0
Other^g^	7 (1)	51.9	9.3–129.2	67.6–512.3	43199	<0.001	0.315	100.0

a *White Line Disease*,

b *White Line Disease and Abscess*,

c *Sole Ulcer*,

d *Sole Ulcer, Sole Haemorrhage/Bruising*,

e *Bovine Digital Dermatitis*,

f *Bovine Digital Dermatitis and Interdigital Phlegmon (foot rot)*,

g *Other hoof related lesions*.

To deal with the high heterogeneity left after the removal of outliers a sub-group analysis was conducted. As with the meta-analysis on prevalence data no sub-group analysis was conducted on the variable *Milking System*.

*Breed, Study Type, Housing Regime, Grazing Regime, Sample Size a*, and *Sample Size b* were identified as predictors for heterogeneity among reported incidence across the different studies with statistical significance (Table 7).

**Table 7:** Sub-group analysis with the papers reporting lameness incidence rate (100 cow-years) at cow level after outlier removal.

**Moderator**	**Subgroup**	**No of studies per subgroup**	**Pooled incidence rate (100 cow-years) per subgroup**	**95% CI**	***P*-value for QM**	**Residual heterogeneity (H)**	**Residual heterogeneity (*I^**2**^*) (%)**
Lameness data source	Mobility scoring system	13	36.9	24.2–52.4	0.989	62.93	100.0
	Records	16	36.8	26.9–48.4			
Study Type	Observational	22	30.7	23.1–39.5	<0.001^*^	63.37	100.0
	Experimental	7	63.4	45.4–84.4			
Study Farm(s) Location	Not at Research Institute	22	33.9	25.8–43.1	0.309	63.57	100.0
	At Research Institute	7	48.3	24.1–80.9			
Breed	Holstein^a^	20	45.7	34.7–58.2	0.0020^*^	45.83	100.0
	Other	6	19.4	9.8–32.4			
Grazing regime	Grazing	13	64.6	43.6–90.1	<0.001^*^	19.66	99.7
	Other	5	17.5	12.2–23.9			
Housing System	Multiple	6	14.8	7.5–24.6	0.03^*^	43.14	99.9
	Cubicle	12	54.2	19.2–106.9			
Calving Pattern	Year-round	5	52.4	18.2–104.2	0.577	43.14	99.9
	Other	9	39.5	22.4–61.3			
Start of data collection (year) 1	1995 and onwards	19	38.1	29.8–47.5	0.694	51.55	100.0
	Before 1995	10	34.6	21.2–51.3			
Start of data collection (year) 2	2000 and onwards	14	43.7	28.5–62.1	0.164	47.84	100.0
	Before 2000	15	31.0	23.3–39.8			
Start of data collection (year) 3	2005 and onwards	9	55.8	28.9–91.5	0.066	45.37	100.0
	Before 2005	20	29.3	23.6–35.6			
Start of data collection (year) 4	2008 and onwards	8	47.8	22.1–83.4	0.316	45.51	100.0
	Before 2008	21	32.9	26.5–40.1			
Start of data collection (year) 5	2010 and onwards	6	31.6	13.7–56.8	0.583	52.07	100.0
	Before 2010	23	38.5	28.9–49.5			
Sample Size a	1,230 animals or more	17	24.5	17.1–33.3	0.0019^*^	62.00	100.0
	less than 1,230 animals	12	60.2	38.3–86.9			
Sample Size b	More than 5 farms and 1230 animals	11	20.7	14.3–28.2	0.0021^*^	43.29	99.9
	less than 5 farms and/or 1,230 animals	18	49.7	31.9–71.6			

a *Herds which cows were mainly Holstein, Friesian and/or Holstein-Friesian*.

* *Variables considered as moderators*.

**Figure 6:**
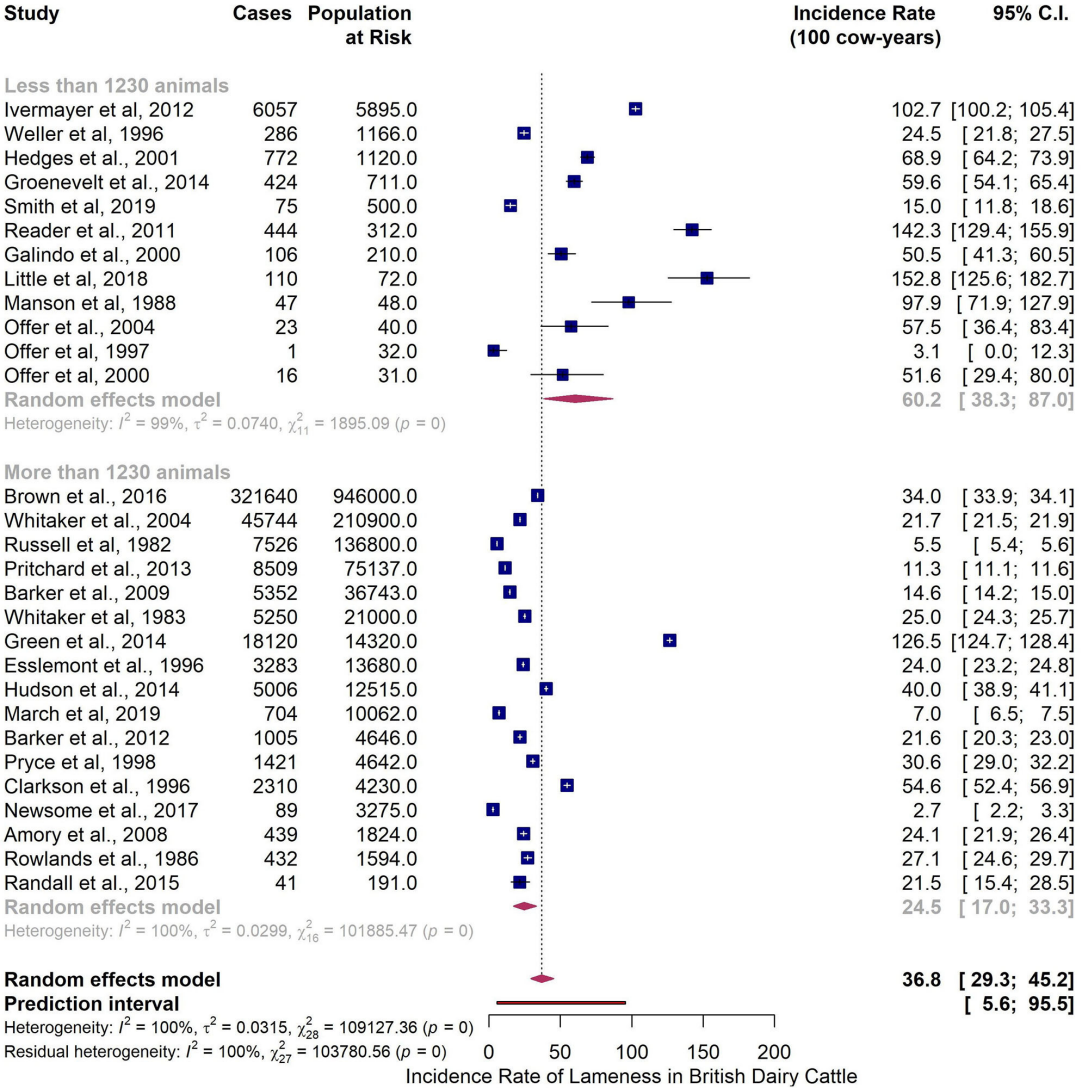
Subgroup analysis of reported incidence rate of lameness in British dairy cattle at cow level with Sample Size a as a moderator.

The six identified predictors were used in the multiple meta-regression model. The most parsimonious model was the one with the moderator *Sample Size a*. The pooled incidence rate of lameness per 100 cow-years for papers based on a sample of 1,230 animals or more was 24.5 (95% CI 17.1–33.3), less than half of the when compared with the pooled estimate from studies based on a sample of <1,230 animals (60.2; 95% CI 38.3–86.9) (Table 7 and Figure 6).

### Risk of Bias

The results of the assessment are presented in Supplementary Table 3 and Supplementary Figure 1 in the Risk Bias Assessment section of the Supplementary Material. Overall all papers were classified at high risk of bias. Even studies that were addressing the review question had sample populations resulting from convenience sampling with high concerns for its effect on the estimate of lameness frequency. The selection of subset of animals within the sample population (e.g., heifers or milking cows) and lameness data source were additional concerns to the introduction of bias (farm records have a recognized issue of under-reporting, and mobility scoring system are subjective by nature).

### Sensitivity Analysis on the Different Data Transformation Methods

Tables with the findings of the sensitivity analysis are provided in Supplementary Tables 6, 7. Below a summary of the main results is presented.

#### Prevalence Data Analysis

The maximum variation in the pooled prevalence when comparing the results between the different models was of about 6% (from 29.9% in with arcsine and double arcsine transformations to 28.1% in the GLM model). The most significant difference was when the lower limits of the 95% prediction intervals were compared: the figure was nearly twice as high with logit transformation (9.9%) when compared with the GLM method (4.9%). Outliers were identified in methods except for the GLM. The most significant difference was in the logit transformation method where the removal of the outliers led to an increase of roughly 3% in the pooled estimate. Heterogeneity was extensive regardless of the method used (Supplementary Table 6).

#### Incidence Data Analysis

Before the removal of outliers the arcsine and double arcsine methods performed quite similarly, and had about 20% more cases per 100 cow-years when compared with the logit transformation. The removal of the outliers translated into a reduction of roughly 8 cases per 100 cow-years in the arcsine and double arcsine methods, and an increase of about 3 cases per 100 cow-years in the logit transformation. After the removal of the identified outliers all three transformation method provided very similar results, with a maximum of 4% variation when comparing the arcsine with the logit method. Heterogeneity was present and extensive irrespective of the data transformation method used (Supplementary Table 7).

## Discussion

The usefulness of outputs from meta-analysis in economic evaluations has been highlighted previously (72, 73). The study performed aimed to generate prevalence and incidence parameters to make assessments of the burden of lameness in British dairy cattle, and to provide a chronological overview of the different lameness detection and classification method and data sources. The analysis indicates that there are problems with how this health condition is reported and measured. It is particularly concerning that 4 out of the 6 studies identified in the SLR published from 2015 onwards based their findings in farm records, a data source highlighted for under-reporting lameness levels (25–28). Additionally the diversity in mobility scoring methods, their intrinsic subjective nature and the potential lack of correspondence adds uncertainty to how consistently is lameness being measured between studies. Other authors have raised this problem (2, 30, 34, 35, 37, 74, 75), there is a need for greater standardization in lameness data collection methods and case definition. This would allow for a more accurate understanding of lameness trends through time and inform the interested parties as to the effectiveness of the adopted approaches for tackling the problem. Despite these problems the analysis has provided great insight into the scientific documentation of lameness and its potential limitations in the estimates of the economic burden of this problem and its serious implication in animal welfare.

We decided to group papers according to the study unit at which lameness was reported, and to concentrate the analysis and report results on cows rather than heifers and culled cows. Heifers are known to have lower incidence of lameness than older cows (76). Additionally culling records are not a good indicator of lameness frequency in dairy cattle, reporting low levels of the ailment (16, 77) when compared with evaluations from independent observers (28, 50). Pooling lameness levels from papers reporting it at heifer and culled cow level with those reporting it at cow level would probably underestimate our results.

The most parsimonious model for the analysis of prevalence data had *Start of data collection (year) 2* as the only moderator. The pooled prevalence for studies for which data began being collected before the year 2000 was 20.0% (95% CI 16.3–24.0%). This is in line with what Clarkson reported (20.6%) in 1996 (78). The two most recent lameness prevalence studies in British dairy cattle reported higher levels of lameness. Griffiths et al. (50) using data collected in 2015 and 2016 estimated a 28.2% prevalence, whereas Randall et al. (79) using data collected in 2014 30.1%. These most current estimates of lameness prevalence in British dairy cattle are similar (although slightly lower) to the pooled estimate for studies for which data began being collected in 2000 and onwards 34.9% (95% CI 30.1–39.9%). It could be that awareness regarding lameness has increased over the years. The fact that the *Start of data collection (year) 4* (year 2008 as cut-off) and *Start of data collection (year) 5* (year 2010 as cut-off) were identified as predictors for the variance in the reported estimates between studies could reflect a higher consciousness to the problem as it was when the British dairy sector adopted the AHDB 4-point MSS as a standard tool in lameness assessment and when the *Healthy Feet* program was launched—two marks in the history of lameness management in the UK (49). Regardless of the potential increase of awareness to the lameness problem in dairy cattle and/or ability to measure it accurately, the frequency levels of this health condition have remained substantially high across time. It is important to acknowledge that the intensification of production system have created pressure on the animal's productivity, sometimes at the expense of their health (4). The selection of animals based solely on milk production has also led to the increase of the incidence of different diseases, namely lameness (80). Lameness has been associated with milk yield: animals with higher milk yields are at higher risk of developing the ailment (81). Yet, early identification of lameness cases and prompt action has proven to be effective in reducing the impact of lameness and maintaining its levels low (82, 83). Developing tools to identify lameness in pre-clinical stages would allow for early intervention providing the necessary support for preventing animals from becoming obviously lame. Genetic improvement of herds based not just on production traits such as milk yield and fertility, but also on resistance to certain health condition such as lameness could offer a way to reduce the incidence of the ailment (84–86). Apart from all the different strategies that can be adopted to alleviate lameness frequency and/or its impact it must be acknowledged that it is up for farmer to make the decisions and take action in managing the health and welfare of the animals. It is thus important to understand the perceptions and motivations of farmers if measures are to be effectively implemented (38, 87).

The moderator *Sample Size a* (1,230 cut-off) retrieved the most parsimonious model when analyzing the lameness incidence data set. The estimated pooled incidence for the studies with more than 1,230 animals indicated 24.5 cases of lameness per 100 cow-years (95% CI 17.1–33.3). This is in line with what Esslemont and Kossaibati (88) and Whitaker et al. (89) estimated−24.5 and 21.8, respectively—but considerably lower than what Clarkson et al. (78) estimated−54.6 cases per 100 cow-years. It must be noted that most studies with a sample size of more than 1,230 animals (12 out of 17) were based on farm records, a data source prone to under-reporting (28, 29). On the other hand 2 out of every 3 studies with a sample size of less 1,230 animals relied on mobility scoring methods to assess lameness. Part of the observed difference could result from the different methods that were used for collecting lameness data. The pooled estimate for incidence rate for studies with <1,230 animals was 60.2 cases of lameness per 100 cow-years (95% CI 38.3–86.9), which is close to what Clarkson et al. (78) estimated. The figure for the incidence in the later study was based on farm records. However, it must be noted that the enrolled farms had regular visits from researchers who mobility scored the herd for the duration of the study, which could have had an effect on the accuracy of the records kept by the farmer.

The impact of lameness is cause-specific, resulting from different adverse effects in the animal's production capacity, and different treatments and prevention and control strategies (17). Identifying the underlying disease leading to lameness is valuable information for the management of hoof problem(s) and for conducting economic studies on this health condition. However, few studies were found to report lameness data with this level of granularity. The fact that collecting such data can be quite time consuming and labor intensive could offer an explanation for this. Dairy farming is time and labor demanding and farmers will have priorities other than to diagnose and register the lameness-causing lesion. Hoof trimmers are an eventual good data source but the pressure to deal with all the animals in a timely manner can lead to misclassification errors and no reporting of such data. In addition to the lack of available data, the diversity in methodologies and sample sizes, and high variance in the reported incidence rate between studies resulted in very wide 95% CI for the pooled estimate.

Data availability and accessibility are bottle-necks when studying animal diseases and their impact (24). The fact that lameness is a symptom rather than a disease in itself (with a diversity of diseases that can cause the ailment), and that different methods are used to capture lameness information, makes data consistency an even higher challenge when studying this health condition (34, 35). Three main findings were drawn from the chronological analysis of lameness detection and classification system: the mobility scoring system (MSS) adopted in 2008 by the industry as the standard (the AHDB Dairy mobility scoring system) is not the only MSS being used for assessing lameness in dairy cattle; the diversity of MSS used and the fact that these are subjective in nature and prone to observer bias makes it more difficult to aim for consistency; and farm records are still a source of data when studying lameness despite the under-reporting problems identified in research (28, 29). The mobility scoring systems are based on ordinal scales, and depend on the observer's experience to detect changes in the animal's locomotion that fit the descriptors for each level in the scale. The scales of the identified MSS ranged from 9 to 4 points with different descriptors. To make the assessments between studies comparable the ordinal scales are translated into binary (lame vs. non-lame) or shorter scales (non-lame, mildly lame, and severely lame). Since the descriptors are not identical between scoring system this could bring about issues of consistency and hamper comparison. Regardless of the myriad of MSS available, further investigation is required to study the impact of the use of different MSS in the reported lameness levels, and to explore how related MSS are between each other. Although technology for the detection of lameness based in artificial intelligence is available only one study has made use of data collected by an automated lameness detection system (ALDS). It must be noted that the validation of ALDS is achieved by comparing the results obtained with the current reference standard—direct observation of the animal. Once parameterized the tool can offer a way to avoid some biases associated with the subjective nature of assessing lameness through direct observation of the animal's behavior and locomotion. However, if the sensitivity was parameterised according to the best available method—MSS—then it is likely that the results from automated system will be influenced by the standard that provided the threshold for lameness condition. Another limitation to ALDS is that some hoof lesions will not alter the animal's behavior or locomotion. This is particularly significant for Bovine Digital Dermatitis, an important infectious hoof disease (75). Although ALDS are a promising tool for objectively identifying lameness there is need for further research in order for it to become a reality. The use of MSS was associated with the study farm(s) location. Research institutes will have implemented a particular system in their routine welfare assessment and thus studies that have made use of their data set for conducting analysis will report this MSS. This could lead to incorrectly concluding that the AHDB system has not been widely adopted if many papers make use of the data set from these dairy farms belonging to research institutes. Nevertheless, and as previously mentioned, diversity in the methods by which lameness is classified brings about inconsistency and hampers comparability. The discussion about the need for University-Industry engagement offer an opportunity to reflect on how to harmonize the tools and communication used by both sectors with respect to lameness in dairy cattle.

The data extracted from the papers was skewed and so it was transformed through the arcsine method to improve its statistical properties. The double arcsine method has been advocated by researchers as the model of choice for conducting meta-analysis with binomial data (53, 55, 90). However, Schwarzer et al. (56) has highlighted potential misleading results from back-transformed data when this method is used, especially when sample size is small. Following Schwarzer et al. (56) recommendations a sensitivity analysis was done to assess the effect of the different data transformation methods on the results. This analysis indicated minor differences in the pooled estimates when using the different methods. The logit transformation was the method that showed most significant differences. Research has indicated this approach to be problematic when analyzing binomial data, placing undue weight on studies reporting extreme proportions and failing to stabilize variance in studies with smaller sample sizes (53, 56, 90). In sum, the arcsine transformation seemed to be the most suitable option.

The choice of a random-effects model seemed appropriate considering the heterogeneity of the methods and sample population between the identified studies, and given the need to consider both the intra and inter-study variance of lameness frequency (54, 55).

With the exception of the GLM model, the DerSimonian and Laird (DL) method was used to estimate the between study variance. Other authors argue that the restricted maximum likelihood (REML) is the approach of choice, despite of higher computational complexity (59). However, it seems that the difference observed when comparing results from these two methods is generally insignificant and its impact negligible (91).

Heterogeneity in the outcome of interest between studies is critical aspect of conducting a meta-analysis (90) and one of its main objectives was to reduce it as much as possible (92). The identified studies were quite diverse in terms of study design, data collection method and analytical approach. This diversity is hard to manage when the number of papers is not big enough to perform the analysis aimed at dealing with heterogeneity. This was particularly marked when exploring the moderator effect of certain factors such as *Grazing Regime, Housing System*, and *Milking System*, for which only a small number of studies had data on. Despite having identified moderators that explained part of the observed heterogeneity, it remained high and unexplained—a common finding when conducting this sort of analysis in disease frequency data (93). As a result of the high residual heterogeneity the interpretation of results should be taken with caution as it may not be appropriate to summarize the data into a single estimate. However, having described the predictors for such heterogeneity is a valuable output from the analysis as these could indicate risk factors for lameness. The estimated prediction intervals are also an important output. While taking into account the variability of individual studies, they are wider than the 95% CI and provide the range of values that would capture 95% of the estimates of lameness frequency levels—meaning that if we were to pick a study on the frequency of lameness in British dairy cattle the figure we would get would fall within that range 95% of the times. These parameters can then be used to inform economic analysis by means of a sensitivity analysis with worst and best case scenarios.

When conducting the meta-analysis on incidence data *Housing Regime* was a factor that seemed to explain part of the observed heterogeneity between studies. These findings are in accordance with conclusions reported in previous publications. Housing system also plays a role in the epidemiology of lameness, with straw yards having a protective effect on lameness incidence (66). The variable *Grazing Regime* was also identified as predictor for the variance of the estimate between studies. As opposed to what research has highlighted (69) grazing systems had a higher incidence rate of lameness (47.5 cases per 100 cow-years; 95% CI 28.8–70.8) when compared with the studies that reported lameness based on a sample that included also included non-grazing systems (17.5 cases per 100 cow-years; 95% CI 12.2–23.9). With the well-documented under-reporting problem of lameness in farm records there was some expectation as to the variable *Lameness Data Source* being identified as a predictor. However, the pooled estimate of the papers with lameness record-base data was not statistically significantly different from the pooled estimate of the papers with lameness data collected through MSS and/or ALDS.

The authors acknowledge that the search terms used in the systematic literature review were somewhat narrow and that some references focusing on lameness in British dairy cattle might have been missed with the search strategy. Yet considering the research question the search terms seem to be adequate when identifying publications focused on reporting lameness frequency levels in British dairy cattle. Limiting the search to research conducted in British dairy cattle was strategic as the results from this analysis are intended to be used in an economic assessment of lameness in this population.

The literature search was restricted to six scientific databases. There is a possibility that some references were not captured in the search. However, these databases were chosen for their extensive coverage of veterinary science journals (94) and thus should have reduced the odds of missing a relevant paper. Although there was no restriction regarding year of publication the retrospective nature of the study might have conditioned data retrieve. Even if authors were still reachable databases were sometime no longer available. Nine papers were not accessible through our methodology. Additionally data could not be retrieved in twelve papers. This could have introduced bias into our analysis, but we do not know the direction of the bias nor the extent due the information lacking.

## Conclusion

Our pooled lameness frequency estimates indicated high levels of the disease with ~30% of British dairy cattle suffering from this ailment at any one moment in time. This analysis will be useful for investigations on the economic impact of lameness on British dairy cattle, by providing information on the burden of lameness, a key parameter for study of Animal Health Economics.

A diversity of detection and classification methods are used for collecting lameness data in the UK. This brings about inconsistency in the existing literature on the subject that hamper results comparison, limiting our ability to see if lameness is changing over time, be it for the purpose of assessing the effectiveness of an intervention or solely for monitoring lameness trend, and to understand lameness impact with precision. The use of artificial intelligence for identifying and monitoring lameness cases could offer objectivity and reliability compared with other lameness detection and classification methods, namely the mobility scoring systems and farm records.

The development and implementation of data collection systems that can offer reliable and standardized information are essential for the decision-making in the realm of animal health management.

## Data Availability Statement

All datasets generated for this study are included in the article/Supplementary Material.

## Author Contributions

The main author was responsible for the systematic literature review, data management and analysis, development of risk bias assessment tools, and for the elaboration of the document. MB was involved in the reference screening and selection and reviewed the manuscript. PK was involved in the development of the code on R software program for the analysis and reviewed the manuscript. HC provided insights on the statistics around meta-analysis and helped guiding the analysis plan. DR contributed with his experience with meta-analysis and guiding the analysis plan. GO contributed with his experience in lameness research and reviewed the document. JR has oversight on the research in general. All co-authors have revised the paper.

## Conflict of Interest

The authors declare that the research was conducted in the absence of any commercial or financial relationships that could be construed as a potential conflict of interest.
