# Supplementary material for: Profiling Detection and Classification of Lameness Methods in British Dairy Cattle Research: A Systematic Review and Meta-Analysis
Source: Front Vet Sci. 2020 Aug 20;7:542. doi: 10.3389/fvets.2020.00542 (PMC7468474; doi:10.3389/fvets.2020.00542)
Supplement: Supplementary file 1 [file Table_1.docx]

# Supplementary material

## Literature review search

The following search terms were used for all six databases: ((dairy AND (cattle OR cow*)) AND (UK OR british OR britain OR kingdom)) AND ((lame* OR locomotion) AND (incidence OR prevalence)). The results from the search and presented in the table below.

Table 1. Scientific databases used in the systematic review and search results

| **Database** | **Website** | **Number of papers** | **Comments on search** |
| --- | --- | --- | --- |
| Agricola | https://agricola.nal.usda.gov/ | 35 | Search on Agricola database and all fields search |
| Cab Direct | https://www.cabdirect.org/ | 325 | All fields search |
| Cochrane library | https://www.cochranelibrary.com/ | 0 | All fields search |
| PubMed | https://pubmed.ncbi.nlm.nih.gov/ | 133 | All fields search |
| Scopus | https://www.scopus.com/ | 78 | Search limited to title, abstract and key words |
| Web of Science | https://webofknowledge.com/ | 176 | Search on all databases and all fields search |

## New variables created

Table 2. New binary variables created for analysing the data extracted from the selected papers for the meta-analysis on lameness frequency levels in British dairy cattle

| **Variable** | **Values** | **Meaning** |
| --- | --- | --- |
| Year of the start of data collection with the year 1995 as a cut-off | Before 1995 | Papers for which data collection started before the year 1995 |
|  | 1995 and onwards | Papers for which data collection started on the year 1995 or after |
| Year of the start of data collection with the year 2000 as a cut-off | Before 2000 | Papers for which data collection started the year 2000 |
|  | 2000 and onwards | Papers for which data collection started on the year 2000 or after |
| Year of the start of data collection with the year 2005 as a cut-off | Before 2005 | Papers for which data collection started the year 2005 |
|  | 2005 and onwards | Papers for which data collection started on the year 2005 or after |
| Year of the start of data collection with the year 2008 as a cut-off | Before 2008 | Papers for which data collection started the year 2008 |
|  | 2008 and onwards | Papers for which data collection started on the year 2008 or after |
| Year of the start of data collection with the year 2010 as a cut-off | Before 2010 | Papers for which data collection started the year 2010 |
|  | 2010 and onwards | Papers for which data collection started on the year 2010 or after |
| Breed of animals | Holstein/Friesian/Holstein-Friesian | Breed of study population is mainly Holstein, Friesian or/and Holstein-Friesians |
|  | Other | All other breeds not fitting the above category |
| Calving pattern | Year-round | Calving pattern of study population is year-round |
|  | Other | All other situations not fitting the above category |
| Grazing regime | Grazing | Study population is allowed to graze during some time of the year |
|  | Other | All other situations not fitting the above category |
| Housing system | Cubicle | Animals are housed in cubicles |
|  | Other | All other situations not fitting the above category |
| Study farm location | Research Institute | Study farm(s) belong to a research institute |
|  | Commercial farm | Study farm(s) are commercial operations |
| Study design | Cross-sectional | Study design was cross-sectional |
|  | Other | All other situations not fitting the above category |
| Study unit | Cow | Study population were dairy cows regardless of their age |
|  | Other | All other situations not fitting the above category |
| Lameness data source | Mobility scoring system | Lameness data were primary collected as animals were mobility scored |
|  | Records | Lameness data derived from records (secondary data source) |
| Sample size with 1230 animals as the cut-off point | 1230 animals or more | Study relied on a sample with 1230 animals or more |
|  | Less than 1230 animals | Study relied on a sample with less than 1230 animals |
| Sample size with 5 farms and 1230 animals as the cut-off point | At least 5 farms and 1230 animals | Study relied on a sample with 1230 animals or more, from 5 or more farms |
|  | Less than 5 farms and/or 1230 animals | Study relied on a sample with less than 1230 animals from less than 5 farms |

## PRISMA-P Checklist

| **Section and topic** | **Item No** | **Checklist item** |  |
| --- | --- | --- | --- |
| **ADMINISTRATIVE INFORMATION** | | |  |
| Title: | | |  |
| Identification | 1a | Systematic Review and Meta-analysis on Lameness Frequency in British Dairy Cattle (article title: Profiling detection and classification of lameness methods in British dairy cattle research: a systematic review and meta-analysis) |  |
| Update | 1b | Not applicable |  |
| Registration | 2 | Nothing to note |  |
| Authors: | | |  |
| Contact | 3a | Joao Sucena Afonso, Department of Epidemiology and Population Health, Institute of Infection and Global Health, University of Liverpool, Liverpool, UK, [jafonso@liverpool.ac.uk](mailto:jafonso@liverpool.ac.uk). |  |
| Contributions | 3b | Joao was responsible for the systematic literature review and meta-analysis plan. Mieghan was involved in the reference screening and selection |  |
| Amendments | 4 | Nothing to note |  |
| Support: | | |  |
| Sources | 5a | Nothing to declare |  |
| Sponsor | 5b | Not applicable |  |
| Role of sponsor or funder | 5c | Not applicable |  |
| **INTRODUCTION** | | |  |
| Rationale | 6 | Lameness is an important health condition in British dairy cattle. However the diversity of study designs, and data collection and analysis methodology used in its research hampers our ability to compare results across different studies making it difficult for people involved in the milk value chain to make informed decisions. |  |
| Objectives | 7 | The approach aimed to answer the question “What is the prevalence and incidence of lameness in British dairy cattle?”. |  |
| **METHODS** | | |  |
| Eligibility criteria | 8 | Eligibility was based on the PICOS approach using the following criteria: (i) Population: British dairy cattle; (ii) Outcome: lameness prevalence and/ or incidence, lameness causing foot lesions; and (iii) Study design: Randomised controlled trials, cohort studies, case-control studies, cross-sectional studies, case reports and outbreak investigations were all eligible for inclusion if they reported number of dairy cows that were lame (numerator) and the study population (denominator), or if the same could be calculated from the reported results. Only studies published in peer-reviewed journals were included, with no date restriction. Language of publication was restricted to English. Papers that reported data from previous publications were excluded as to have only one entry per data collection exercise. If the study met all the inclusion criteria but did not provide data on the number of lame cows and/or study population the corresponding author was contacted via email in an effort to retrieve the missing information and for clarification. If the corresponding author was not available, one of the co-authors was contacted. If the author(s) did not reply or could not provide the information requested the paper was excluded from the meta-analysis. In addition to the references identified through the systematic review, a backward search (also known as chain search) was also conducted and if papers were eligible were added to the database. |  |
| Information sources | 9 | The search was conducted in six electronic scientific literature databases – *Agricola*, *Cab* *Direct*, *Cochrane* *Library*, *PubMed*, *Scopus* and *Web* *of* *Science* (all databases) on the 4th of January 2020 with no date restriction |  |
| Search strategy | 10 | The search was limited to peer reviewed articles, published since 1823 in English. The population search terms were (dairy AND cattle) AND (UK OR Britain OR British OR kingdom). The outcome search terms were (lameness AND (prevalence OR incidence). The following code was used for all six databases considered: (dairy AND (cattle OR cow*) AND (UK OR British OR Britain OR kingdom) AND (lame* OR locomotion) AND (incidence OR prevalence) |  |
| Study records: | | |  |
| Data management | 11a | EndNote X9 was used to manage citations. Duplicate entries were identified, using the automatic function in EndNote and manually during the screening process, by considering the author, the year of publication, the article title, and the volume, issue and page numbers of the source. In questionable cases, the abstracts or full texts were compared. Conference papers reporting studies that were subsequently published in journals were considered duplicates |  |
| Selection process | 11b | Titles and abstracts from the records identified in the search were screened and accepted for full-text screening (eligibility) based on the inclusion criteria sited above by the main author. Full-text screening was performed by one reviewer (JSA) and checked for accuracy by MB. Any ambiguities were discussed and consensus reached | |
| Data collection process | 11c | Data extraction was conducted by one reviewer (JSA) and validated by another (MB) | |
| Data items | 12 | Study characteristics (authors, year of publication, year or years of data collection, study type - experimental or observational, study design, sample size, sampling strategy); (ii) population data (breed, production system, milking system, grazing regime, housing system, study unit); (iii) outcome data (lameness classification method, lameness assessment frequency, lameness assessment observer, measure of disease frequency); and (iv) numerator and denominator data (number of lame cows, total number of cows in the study population, number of lameness events, population at risk | |
| Outcomes and prioritization | 13 | The primary outcome of interest was lameness frequency (incidence rate and prevalence) | |
| Risk of bias in individual studies | 14 | The lameness frequency levels reported in the papers included for the meta-analysis were assessed as to their potential risk of bias. This exercise followed the QUADAS2 approach ([1](#_ENREF_1))([Broen, Braaksma et al. 2012](#_ENREF_11))([Broen, Braaksma et al. 2012](#_ENREF_11))and an adapted tool was used to evaluate the potential risk of bias of a set of components and its applicability. The tool was piloted by two researchers (JSA and an invited researcher – BG - who was not otherwise involved in the study) on two randomly selected papers. If there was no agreement between the two researchers when assessing the papers, the tool was revised and re-piloted on two other randomly selected papers. A paper was considered to have a low overall risk of bias if the risk of bias and applicability concerns were low | |
| Data synthesis | 15a | The primary outcome measure was incidence rate or prevalence of lameness. The data reported in the studies was summarised in a pooled estimate of incidence rate or prevalence of lameness | |
|  | 15b | A random effects model was used to pool the estimates from the identified studies. As data was not normally distributed it was transformed using the arcsine transformation method. The inverse variance method was used for pooling the estimate of the lameness frequency level. Confidence intervals for individual studies were estimated through the normal approximation interval based on the summary measure. The DerSimonian-Laird (DL) estimate was used to calculate the between-study variance *τ^2^* | |
|  | 15c | A sensitivity analysis was conducted comparing the results obtained using the arcsine transformation with those obtained when using other available data transformation methods.  A two-step approach was used to address heterogeneity. The first-step was to identify outliers and influential studies. The forest plot was assessed and studies whose 95% confidence intervals did not overlap with that from the pooled estimate were identified. A set of tests followed to formally assess the influence of the outlying effect of individual studies on the pooled estimate by means of the function *influence.* Papers that had a strong influence on the overall estimate were removed from the meta-analysis. The second step was to use a moderator analysis, first by sub-group analysis (univariate), grouping the studies by factors that could explain the heterogeneity, followed by a multiple meta-regression if more than one factor was identified as a predictor of the variance between studies. Factors providing a P-value of 0.1 or below in the test for moderators were considered moderators and added to the multiple meta-regression model. The second step was only conducted if there were at least 10 papers, and if there were at least 5 papers per subgroup. | |
|  | 15d | Nothing to note | |
| Meta-bias(es) | 16 | Nothing to note | |
| Confidence in cumulative evidence | 17 | Nothing to note | |

## Risk Bias Assessment

### QUADAS-2 adapted tool ([1](#_ENREF_1))

QUADAS-2 – adapted tool for assessing the risk of bias of selected publications for the meta-analysis on lameness frequency in British dairy cattle

| **Phase 1 – Review Question** |
| --- |
| What is the frequency level of lameness in British dairy cattle? |

| **Phase 2 – Risk of Bias** | | | |
| --- | --- | --- | --- |
| 1. Selection of Study Population | | | |
| Farm sampling strategy | Randomly sampled from whole study population | | (Low Risk) |
|  | Randomly sampled from subset of the whole study population | | (High Risk) |
|  | Convenience sampled | | (High Risk) |
|  | Unclear | | (Unknown) |
|  | Comments: | | |
| Animal sampling strategy | Whole herd evaluated | | (Low Risk) |
|  | Random sample of the herd evaluated | | (High Risk) |
|  | Convenience/subset sample of the herd evaluated | | (High Risk) |
|  | Unclear | | (Unknown) |
|  | Comments: | | |
| Refusal to participate or dropouts | No farmers refused to participate in the study nor did they dropout from the study once enrolled | | (Low Risk) |
|  | There were refusals to participate in the study and/or dropouts | | (High Risk) |
|  | Unclear | | (Unknown) |
|  | Comments: | | |
| Risk of Bias | If any of the answers for the different points was High Risk the Risk of Bias is considered High | | (Low Risk) |
|  |  |  | (High Risk) |
| 2. Study’s Objective | | | |
| Study’s primary objective was to estimate the frequency level of lameness | | | (Low Risk) |
| Study’s primary objective was NOT to estimate the frequency level of lameness | | | (High Risk) |
| Unclear | | | (Unknown) |
| Comments: | | | |
| 3. Collection/Source of Lameness Data | | | |
| Lameness data source/collection method  Applicability Judgement | By the same trained investigator through a mobility scoring system | | (Low Risk) |
|  | By two or more trained investigators using the same mobility scoring system | The inter-observer agreement is high | (Low Risk) |
|  |  | The inter-observer agreement is moderate or low | (High Risk) |
|  | Lameness data retrieved from farm records | | (High Risk) |
|  | Lameness data retrieved from veterinary/hoof trimmer records | | (Low Risk) |
|  | Assessed by automated lameness detection system | | (Low Risk) |
|  | Unclear | | (Unknown) |
|  | Comments: | | |

| **Phase 3 – Bias Applicability Judgment** | | |
| --- | --- | --- |
| 1. Selection of Study Population | | |
| Has the selection of the study population significantly affected its representativeness of the target population? | Yes | (Low Risk) |
|  | No | (High Risk) |
|  | Unclear | (Unknown) |
|  | Comments: | |
| 2. Study’s Objective | | |
| Is the study addressing the review question? | Yes | (Low Risk) |
|  | No | (High Risk) |
|  | Unclear | (Unknown) |
|  | Comments: | |
| 3. Collection/Source of Lameness Data | | |
| Has lameness data been collected in an objective and consistent way? | Yes | (Low Risk) |
|  | No | (High Risk) |
|  | Unclear | (Unknown) |
|  | Comments: | |

| **Phase 4 – Overall Risk Assessment** |
| --- |
| Overall Risk is considered LOW if Risk of Bias and Applicability Judgment are LOW for all criteria. All other situation are considered are classified as HIGH |

### Risk of bias assessment results

Table 3. Summary results of the risk of bias assessment using the adapted QUADAS-2 tool

| **Study** | **Risk of bias** | | |  | **Applicability concerns** | | | **Overall risk assessment** |
| --- | --- | --- | --- | --- | --- | --- | --- | --- |
|  | **Selection of study population** | **Study’s objective** | **Collection/source of lameness data** |  | **Selection of study population** | **Study’s objective** | **Collection/source of lameness data** |  |
| ([2](#_ENREF_2)) | High | Low | Low |  | High | Low | Low | High |
| ([3](#_ENREF_3)) | High | Low | Low |  | High | Low | Low | High |
| ([4](#_ENREF_4)) | High | Low | Low |  | High | Low | Low | High |
| ([5](#_ENREF_5)) | High | Low | Low |  | High | Low | Low | High |
| ([6](#_ENREF_6)) | High | High | Low |  | High | Low | Low | High |
| ([7](#_ENREF_7)) | High | Low | High |  | High | Low | High | High |
| ([8](#_ENREF_8)) | High | Low | Low |  | High | Low | Low | High |
| ([9](#_ENREF_9)) | High | Low | Low |  | High | Low | Low | High |
| ([10](#_ENREF_10)) | High | Low | Low |  | High | Low | High | High |
| ([11](#_ENREF_11)) | High | Low | Low |  | High | Low | Low | High |
| ([12](#_ENREF_12)) | High | Low | High |  | High | Low | High | High |
| ([13](#_ENREF_13)) | High | High | Low |  | High | High | Low | High |
| ([14](#_ENREF_14)) | High | Low | Low |  | High | Low | Low | High |
| ([15](#_ENREF_15)) | High | High | High |  | High | Low | Low | High |
| ([16](#_ENREF_16)) | High | Low | High |  | High | Low | High | High |
| ([17](#_ENREF_17)) | High | High | High |  | High | High | High | High |
| ([18](#_ENREF_18)) | High | Low | Low |  | High | Low | Low | High |
| ([19](#_ENREF_19)) | High | Low | Low |  | High | Low | Low | High |
| ([20](#_ENREF_20)) | High | Low | High |  | High | Low | Low | High |
| ([21](#_ENREF_21)) | High | Low | Low |  | High | Low | Low | High |
| ([22](#_ENREF_22)) | High | Low | Low |  | High | Low | Low | High |
| ([23](#_ENREF_23)) | High | Low | Low |  | High | Low | Low | High |
| ([24](#_ENREF_24)) | High | High | Low |  | High | High | Low | High |
| ([25](#_ENREF_25)) | High | Low | High |  | High | Low | Low | High |
| ([26](#_ENREF_26)) | High | Low | High |  | High | Low | High | High |
| ([27](#_ENREF_27)) | High | Low | High |  | High | Low | High | High |
| ([28](#_ENREF_28)) | High | Low | Low |  | High | Low | Low | High |
| ([29](#_ENREF_29)) | High | Low | Low |  | High | Low | Low | High |
| ([30](#_ENREF_30)) | High | Low | Low |  | High | Low | Low | High |
| ([31](#_ENREF_31)) | High | Low | High |  | High | Low | Low | High |
| ([32](#_ENREF_32)) | High | Low | High |  | High | Low | High | High |
| ([33](#_ENREF_33)) | High | Low | Low |  | High | Low | Low | High |
| ([34](#_ENREF_34)) | High | Low | High |  | High | Low | High | High |
| ([35](#_ENREF_35)) | High | High | Low |  | High | Low | Low | High |
| ([36](#_ENREF_36)) | High | Low | High |  | High | Low | High | High |
| ([37](#_ENREF_37)) | High | Low | High |  | High | Low | High | High |
| ([38](#_ENREF_38)) | High | Low | Low |  | High | Low | Low | High |
| ([39](#_ENREF_39)) | High | Low | High |  | High | Low | High | High |
| ([40](#_ENREF_40)) | High | Low | Low |  | High | Low | Low | High |
| ([41](#_ENREF_41)) | High | High | High |  | High | Low | Low | High |
| ([42](#_ENREF_42)) | High | Low | Low |  | High | Low | Low | High |
| ([43](#_ENREF_43)) | High | High | Low |  | High | High | Low | High |
| ([44](#_ENREF_44)) | High | High | Low |  | High | Low | Low | High |
| ([45](#_ENREF_45)) | High | Low | Low |  | High | Low | Low | High |
| ([46](#_ENREF_46)) | High | Low | Low |  | High | Low | Low | High |
| ([47](#_ENREF_47)) | High | Low | Low |  | High | Low | Low | High |
| ([48](#_ENREF_48)) | High | High | High |  | High | High | High | High |
| ([49](#_ENREF_49)) | High | High | Low |  | High | High | Low | High |
| ([50](#_ENREF_50)) | High | Low | Low |  | High | Low | Low | High |
| ([51](#_ENREF_51)) | High | Low | High |  | High | Low | High | High |
| ([52](#_ENREF_52)) | High | Low | High |  | High | Low | High | High |
| ([53](#_ENREF_53)) | High | Low | Low |  | High | Low | Low | High |
| ([54](#_ENREF_54)) | High | Low | High |  | High | Low | Low | High |
| ([55](#_ENREF_55)) | High | Low | Low |  | High | Low | Low | High |
| ([56](#_ENREF_56)) | High | Low | Low |  | High | Low | Low | High |
| ([57](#_ENREF_57)) | High | Low | High |  | High | Low | Low | High |
| ([58](#_ENREF_58)) | High | Low | High |  | High | Low | High | High |
| ([59](#_ENREF_59)) | High | Low | Low |  | High | Low | Low | High |
| ([60](#_ENREF_60)) | High | Low | Low |  | High | Low | Low | High |
| ([61](#_ENREF_61)) | High | Low | Low |  | High | Low | Low | High |
| ([62](#_ENREF_62)) | High | Low | Low |  | High | Low | Low | High |
| ([63](#_ENREF_63)) | High | Low | Low |  | High | Low | Low | High |
| ([64](#_ENREF_64)) | High | Low | High |  | High | Low | High | High |
| ([65](#_ENREF_65)) | High | Low | High |  | High | Low | High | High |
| ([66](#_ENREF_66)) | High | Low | High |  | High | Low | High | High |
| ([67](#_ENREF_67)) | High | Low | High |  | High | Low | High | High |
| ([68](#_ENREF_68)) | High | Low | High |  | High | Low | High | High |
| ([69](#_ENREF_69)) | High | Low | High |  | High | Low | High | High |


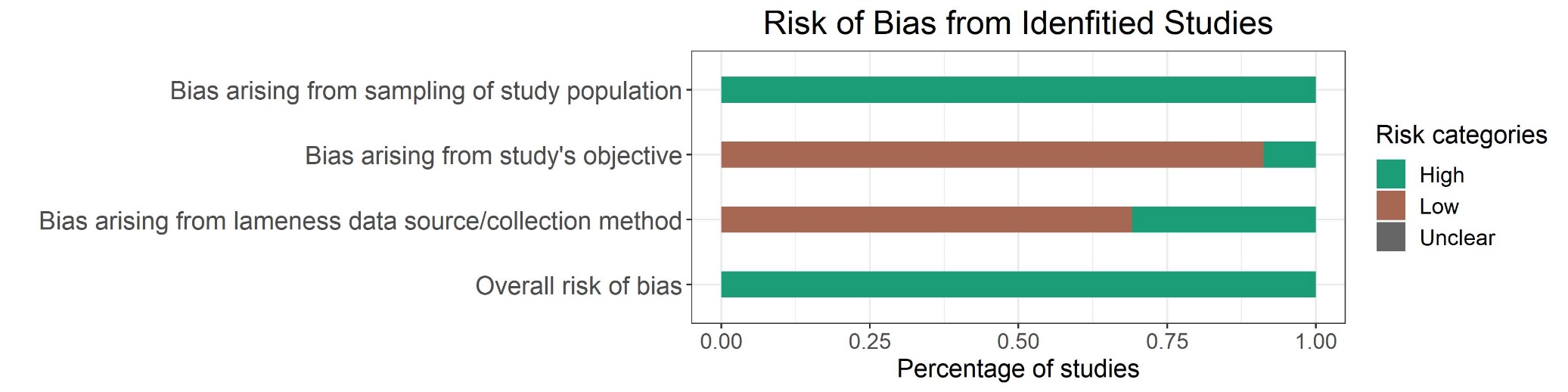


Figure 1. Diagram of risk of bias from identified studies

## Papers identified for the meta-analysis

Table 4. Summary table of characteristics of papers with prevalence data selected for the meta-analysis

| Author and year | Study type | Study design | Study farm(s) at Research Institute? | Study unit | Lameness data source | Farm sample size | Animal Sample size | No of lame animals |
| --- | --- | --- | --- | --- | --- | --- | --- | --- |
| ([2](#_ENREF_2)) | Observational | Longitudinal | No | Cow | Farm records | 30 | 1824 | 636 |
| ([3](#_ENREF_3)) | Observational | Longitudinal | No | Cow | 4-point scale AHDB DairyCo 2008 | 7 | 1400 | 868 |
| ([5](#_ENREF_5)) | Observational | Cross-sectional | No | Cow | 4-point scale Whay et al 2003 | 205 | 33415 | 12297 |
| ([6](#_ENREF_6)) | Observational | Longitudinal | No | Cow | 3-point scale Amory et al 2006 | 50 | 4646 | 3458 |
| ([8](#_ENREF_8)) | Observational | Longitudinal | No | Cow | Automated system | 1 | 332 | 40 |
| ([9](#_ENREF_9)) | Observational | Longitudinal | No | Cow | 5-point scale Flower and Weary 2006 | 1 | 25 | 5 |
| ([10](#_ENREF_10)) | Observational | Longitudinal | No | Cow | None | 97 | 1008 | 39 |
| ([11](#_ENREF_11)) | Observational | Longitudinal | Yes | Heifer | 9-point scale Manson and Leaver 1988 | 1 | 513 | 14 |
| ([12](#_ENREF_12)) | Observational | Retrospective longitudinal | No | Cow | Farm records | Not reported | 946000 | 166496 |
| ([13](#_ENREF_13)) | Experimental | Longitudinal | Yes | Cow | 9-point scale Manson and Leaver 1988 | 2 | 178 | 13 |
| ([14](#_ENREF_14)) | Observational | Longitudinal | No | Cow | 9-point scale Manson and Leaver 1988 | 37 | 4230 | 871 |
| ([15](#_ENREF_15)) | Experimental | Longitudinal | No | Cow | Farm records | 1 | 10 | 1 |
| ([16](#_ENREF_16)) | Observational | Retrospective longitudinal | No | Cow | Vet and farm records | 90 | 13680 | 2380 |
| ([17](#_ENREF_17)) | Observational | Retrospective longitudinal | No | Culled cows | Farm records | 50 | 26644 | 418 |
| ([18](#_ENREF_18)) | Experimental | Longitudinal | Yes | Heifer | 9-point scale Manson and Leaver 1988 | 1 | 80 | 32 |
| ([19](#_ENREF_19)) | Observational | Longitudinal | No | Cow | 5-point scale Galindo et al 2000 | 3 | 210 | 42 |
| ([21](#_ENREF_21)) | Observational | Cross-sectional | No | Cow | 4-point scale AHDB DairyCo 2008 | 61 | 14700 | 4145 |
| ([22](#_ENREF_22))* | Experimental | Longitudinal | No | Cow | 4-point scale AHDB DairyCo 2008 | 4 | 711 | 271 |
| ([23](#_ENREF_23)) | Observational | Cross-sectional | No | Cow | 5-point scale Haskell MJ et al 2006 | 37 | 2724 | 647 |
| ([27](#_ENREF_27)) | Observational | Retrospective longitudinal | No | Lactation | Farm records | 960 | 63891 | 2429 |
| ([29](#_ENREF_29)) | Experimental | Longitudinal | No | Cow | 4-point scale AHDB DairyCo 2008 | 4 | 680 | 318 |
| ([30](#_ENREF_30)) | Observational | Longitudinal | No | Cow | 4-point scale Whay et al 2003 | 4 | 731 | 209 |
| ([31](#_ENREF_31)) | Experimental | Longitudinal | Yes | Cow | Farm records | 1 | 53 | 35 |
| ([32](#_ENREF_32)) | Experimental | Longitudinal | Yes | Cow | Farm records | 1 | 72 | 42 |
| ([33](#_ENREF_33)) | Experimental | Negatively controlled randomized clinical trial | No | Heifer | 6-point scale Thomas HJ et al 2015 | 1 | 418 | 174 |
| ([34](#_ENREF_34)) | Observational | Retrospective longitudinal | No | Cow | Farm records | 9 | 224 | 73 |
| ([35](#_ENREF_35)) | Experimental | Longitudinal | Yes | Cow | 9-point scale Manson and Leaver 1988 | 1 | 48 | 15 |
| ([38](#_ENREF_38)) | Experimental | Negatively controlled randomized clinical trial | No | Heifer | 4-point scale AHDB DairyCo 2008 | 8 | 281 | 48 |
| ([39](#_ENREF_39)) | Observational | Cross-sectional | No | Cow | 9-point scale Manson and Leaver 1988 | 15 | 1575 | 82 |
| ([40](#_ENREF_40)) | Observational | Longitudinal | No | Cow | 5-point scale Sprecher et al 1997 | 2 | 74 | 42 |
| ([41](#_ENREF_41)) | Observational | Longitudinal | No | Cow | Farm records | 4 | 1307 | 521 |
| ([42](#_ENREF_42)) | Observational | Retrospective Longitudinal | Yes | Culled cows | 9-point scale Manson and Leaver 1988 | 1 | 72 | 8 |
| ([44](#_ENREF_44)) | Experimental | Longitudinal | Yes | Cow | 9-point scale Manson and Leaver 1988 | 1 | 32 | 3 |
| ([46](#_ENREF_46)) | Experimental | Longitudinal | Yes | Heifer | 9-point scale Manson and Leaver 1988 | 1 | 20 | 10 |
| ([47](#_ENREF_47)) | Experimental | Longitudinal | Yes | Cow | 9-point scale Manson and Leaver 1988 | 1 | 40 | 9 |
| ([48](#_ENREF_48)) | Observational | Retrospective Longitudinal | No | Culled cows | Farm records | 843 | 133910 | 5337 |
| ([49](#_ENREF_49)) | Experimental | Longitudinal | Yes | Cow | 4-point scale Phillips, C. J. C. 2009 | 1 | 162 | 98 |
| ([50](#_ENREF_50)) | Observational | Cross-sectional | No | Cow | 4-point scale Whay et al 2003 | 63 | 3390 | 1217 |
| ([51](#_ENREF_51)) | Observational | Longitudinal | No | Lactation | Farm records | 2434 | 44449 | 7099 |
| ([53](#_ENREF_53))* | Observational | Longitudinal | Yes | Cow | 9-point scale Manson and Leaver 1988 | 1 | 724 | 147 |
| ([55](#_ENREF_55)) | Observational | Cross-sectional | No | Cow | 4-point scale AHDB DairyCo 2008 | 43 | 5620 | 1692 |
| ([56](#_ENREF_56)) | Observational | Longitudinal | No | Cow | 4-point scale Whay et al 2003 | 1 | 312 | 100 |
| ([59](#_ENREF_59)) | Observational | Cross-sectional | No | Cow | 4-point scale Rutherford et al 2009 | 80 | 12100 | 2334 |
| ([60](#_ENREF_60)) | Observational | Longitudinal | No | Cow | 4-point scale AHDB DairyCo 2008 | 2 | 500 | 103 |
| ([61](#_ENREF_61)) | Experimental | Positively controlled randomized clinical trial (RCT) | No | Cow | 6-point scale Thomas HJ et al 2015 | 7 | 648 | 176 |
| ([62](#_ENREF_62)) | Observational | Longitudinal | No | Cow | 3-point scale Walker et al 2007 | 1 | 36 | 18 |
| ([63](#_ENREF_63)) | Observational | Longitudinal | No | Cow | 3-point scale Walker et al 2007 | 1 | 59 | 39 |
| ([64](#_ENREF_64)) | Observational | Retrospective Longitudinal | No | Cow | Farm records | 55 | 7700 | 2310 |
| ([67](#_ENREF_67)) | Observational | Retrospective Longitudinal | No | Cow | Farm records | 340 | 45220 | 10717 |
| ([69](#_ENREF_69)) | Observational | Retrospective Longitudinal | No | Cow | Farm records | 1 | 150 | 102 |
| ^*^ data supplied by author | | | | | | | | |

Table 5. Summary table of characteristics of papers with incidence data selected for the meta-analysis

| Author and year | Study type | Study design | Study farm(s) at Research Institute? | Study unit | Lameness data source | Farm sample size | Animal Sample size | No of lameness events |
| --- | --- | --- | --- | --- | --- | --- | --- | --- |
| ([2](#_ENREF_2)) | Observational | Longitudinal | No | Cow | Farm records | 30 | 1824 | 439 |
| ([4](#_ENREF_4)) | Observational | Retrospective Longitudinal | No | Cow | 3-point scale Amory et al 2006 | 28 | 3154 | 446 |
| ([6](#_ENREF_6)) | Observational | Longitudinal | No | Cow | 3-point scale Amory et al 2006 | 50 | 4646 | 1005 |
| ([7](#_ENREF_7)) | Observational | Retrospective Longitudinal | No | Cow | Vet records | 1 | 431 | 1638 |
| ([12](#_ENREF_12)) | Observational | Retrospective Longitudinal | No | Cow | Farm records | Not reported | 946000 | 321640 |
| ([14](#_ENREF_14)) | Observational | Longitudinal | No | Cow | 9-point scale Manson and Leaver 1988 | 37 | 4230 | 2310 |
| ([16](#_ENREF_16)) | Observational | Retrospective Longitudinal | No | Cow | Vet and farm records | 90 | 13680 | 3283 |
| ([18](#_ENREF_18)) | Experimental | Longitudinal | Yes | Heifer | 9-point scale Manson and Leaver 1988 | 1 | 80 | 25 |
| ([19](#_ENREF_19)) | Observational | Longitudinal | No | Cow | 5-point scale Galindo et al 2000 | 3 | 210 | 106 |
| ([20](#_ENREF_20)) | Observational | Longitudinal | No | Cow | Farm records | 1 | 14320 | 18120 |
| ([22](#_ENREF_22)) | Experimental | Longitudinal | No | Cow | 4-point scale AHDB DairyCo 2008 | 4 | 711 | 424 |
| ([24](#_ENREF_24)) | Experimental | Longitudinal | No | Cow | Vet and farm records | 5 | 1120 | 772 |
| ([25](#_ENREF_25)) | Observational | Retrospective Longitudinal | No | Lactation | Vet records | 39 | 12515 | 5006 |
| ([26](#_ENREF_26)) | Observational | Longitudinal | No | Cow | Farm records | 15 | 5895 | 6057 |
| Leach ([28](#_ENREF_28))et al., 2005 | Experimental | Longitudinal | Yes | Heifer | 9-point scale Manson and Leaver 1988 | 1 | 1356 | 40 |
| ([32](#_ENREF_32)) | Experimental | Longitudinal | Yes | Cow | Farm records | 1 | 72 | 110 |
| ([35](#_ENREF_35)) | Experimental | Longitudinal | Yes | Cow | 9-point scale Manson and Leaver 1988 | 1 | 48 | 47 |
| ([36](#_ENREF_36)) | Observational | Retrospective Longitudinal | No | Cow | Farm records | 43 | 10062 | 704 |
| ([37](#_ENREF_37)) | Observational | Retrospective Longitudinal | No | Culled cow | Farm records | 96 | 17053 | 887 |
| ([43](#_ENREF_43)) | Observational | Longitudinal | No | Cow | 6-point scale Thomas HJ et al 2015 | 2 | 3275 | 89 |
| ([44](#_ENREF_44)) | Experimental | Longitudinal | Yes | Cow | 9-point scale Manson and Leaver 1988 | 1 | 32 | 3 |
| ([45](#_ENREF_45)) | Experimental | Longitudinal | Yes | Cow | 9-point scale Manson and Leaver 1988 | 1 | 31 | 16 |
| ([46](#_ENREF_46)) | Experimental | Longitudinal | Yes | Heifer | 9-point scale Manson and Leaver 1988 | 1 | 20 | 17 |
| ([47](#_ENREF_47)) | Experimental | Longitudinal | Yes | Cow | 9-point scale Manson and Leaver 1988 | 1 | 40 | 9 |
| ([51](#_ENREF_51)) | Observational | Longitudinal | No | Lactation | Farm records | 2434 | 75137 | 8509 |
| ([52](#_ENREF_52)) | Observational | Longitudinal | No | Cow | Farm records |  | 4642 | 1421 |
| ([53](#_ENREF_53)) | Observational | Longitudinal | Yes | Cow | 9-point scale Manson and Leaver 1988 | 1 | 191 | 41 |
| ([54](#_ENREF_54)) | Observational | Retrospective Longitudinal | Yes | Heifer | 9-point scale Manson and Leaver 1988 | 1 | 145 | 552 |
| ([56](#_ENREF_56)) | Observational | Longitudinal | No | Cow | 4-point scale Whay et al 2003 | 1 | 312 | 444 |
| ([57](#_ENREF_57)) | Observational | Longitudinal | Yes | Lactation | Farm records | 4 | 1594 | 432 |
| ([58](#_ENREF_58)) | Observational | Longitudinal | No | Cow | Vet records | 1821 | 136800 | 7526 |
| ([60](#_ENREF_60)) | Observational | Longitudinal | No | Cow | 4-point scale AHDB DairyCo 2008 | 2 | 500 | 75 |
| ([65](#_ENREF_65)) | Observational | Longitudinal | No | Cow | Farm records | 11 | 1166 | 286 |
| ([66](#_ENREF_66)) | Observational | Retrospective Longitudinal | No | Cow | Vet records | 185 | 21000 | 5250 |
| ([68](#_ENREF_68)) | Observational | Retrospective Longitudinal | No | Cow | Farm records | 434 | 210900 | 45744 |
| ([69](#_ENREF_69)) | Observational | Retrospective Longitudinal | No | Cow | Farm records | 1 | 150 | 242 |

## Results from the sensitivity analysis on the different data transformation methods

Table 6. Summary of the results from the meta-analysis of studies reporting lameness prevalence at cow level using different data transformation methods, before and after outlier removal

| **Transformation** | **No of studies** | **Pooled prevalence** | **95% CI** | | **95% PI** | **Heterogeneity measures** | | | |
| --- | --- | --- | --- | --- | --- | --- | --- | --- | --- |
|  |  |  |  |  |  | **Cochran’s Q** | **P-value Q** | **Tau^2^** | ***I^2^*** |
| Before outlier identification and removal | | | | | | | | | |
| Arcsine | 42 | 0.299 | 0.261-0.339 | 0.087-0.572 | | 34975 | <0.001 | 0.018 | 99.9% |
| Double arcsine | 42 | 0.299 | 0.260-0.339 | 0.085-0.575 | | 34971 | <0.001 | 0.019 | 99.9% |
| GLMM | 42 | 0.281 | 0.222-0.349 | 0.049-0.747 | | 33164 | <0.001 | 1.039 | 99.9% |
| Logit | 42 | 0.285 | 0.246-0.327 | 0.099-0.592 | | 27320 | <0.001 | 0.399 | 99.8% |
| After outlier identification and removal | | | | | | | | | |
| Arcsine | 40 | 0.295 | 0.267-0.324 | 0.138-0.482 | | 12892 | <0.001 | 0.009 | 99.7% |
| Double arcsine | 40 | 0.294 | 0.266-0.323 | 0.135-0.483 | | 12890 | <0.001 | 0.009 | 99.7% |
| GLMM | 42^*^ | - | - | - | | - | - | - | - |
| Logit | 38 | 0.312 | 0.279-0.348 | 0.147-0.545 | | 14013 | <0.001 | 0.222 | 99.7% |

^*^no outliers identified

Table 7. Summary of the results from the meta-analysis of studies reporting lameness incidence rate (100 cow-years) at cow level using different data transformation methods, before and after outlier removal

| **Transformation** | **No of studies** | **Pooled Incidence rate (100 cow-years)** | **95% CI** | | **95% PI** | **Heterogeneity measures** | | | |
| --- | --- | --- | --- | --- | --- | --- | --- | --- | --- |
|  |  |  |  |  |  | **Cochran’s Q** | **P-value Q** | **Tau^2^** | ***I^2^*** |
| Before outlier identification and removal | | | | | | | | | |
| Arcsine | 31 | 45.2 | 36.9-54.3 | 8.8-109.7 | | 112985 | <0.001 | 0.033 | 100.0% |
| Double arcsine | 31 | 45.2 | 36.9-54.3 | 8.7-109.9 | | 112982 | <0.001 | 0.033 | 100.0% |
| Logit | 31 | 35.2 | 27.1-45.6 | 7.8-159.4 | | 102689 | <0.001 | 0.528 | 100.0% |
| After outlier identification and removal | | | | | | | | | |
| Arcsine | 29 | 36.8 | 29.3-45.3 | 5.6-95.5 | | 109127 | <0.001 | 0.032 | 100.0% |
| Double arcsine | 29 | 36.9 | 29.3-45.3 | 5.5-95.7 | | 109121 | <0.001 | 0.032 | 100.0% |
| Logit | 28 | 38.1 | 30.1-48.0 | 10.5-137.9 | | 68058 | <0.001 | 0.378 | 100.0% |

## References

1. Broen MP, Braaksma MM, Patijn J, Weber WE. Prevalence of pain in Parkinson's disease: a systematic review using the modified QUADAS tool. Movement disorders : official journal of the Movement Disorder Society. 2012;27(4):480-4.

2. Amory JR, Barker ZE, Wright JL, Mason SA, Blowey RW, Green LE. Associations between sole ulcer, white line disease and digital dermatitis and the milk yield of 1824 dairy cows on 30 dairy cow farms in England and Wales from February 2003–November 2004. Preventive Veterinary Medicine. 2008;83(3):381-91.

3. Archer SC, Green MJ, Huxley JN. Association between milk yield and serial locomotion score assessments in UK dairy cows. Journal of Dairy Science. 2010;93(9):4045-53.

4. Barker Z, Amory J, Wright J, Mason S, Blowey R, Green L. Risk factors for increased rates of sole ulcers, white line disease, and digital dermatitis in dairy cattle from twenty-seven farms in England and Wales. Journal of dairy science. 2009;92:1971-8.

5. Barker ZE, Leach KA, Whay HR, Bell NJ, Main DCJ. Assessment of lameness prevalence and associated risk factors in dairy herds in England and Wales. Journal of Dairy Science. 2010;93(3):932-41.

6. Barker Z, Wright JL, Blowey R, Amory J, Green L. Uptake and effectiveness of interventions to reduce claw lesions in 40 dairy herds in the UK. Animal Welfare. 2012;21(4):563-76.

7. Bell NI. Using foot lesion prevalence data in an investigation of lameness associated with palm kernel in a dairy blend. UK Vet: Livestock. 2012;17(2):37...41.

8. Bell NJ. Case report: reducing lameness through active screening, attention to details and skilled staff. Summa, Animali da Reddito. 2013;8(3):39-46.

9. Blackie N, Maclaurin L. Influence of Lameness on the Lying Behaviour of Zero-Grazed Lactating Jersey Dairy Cattle Housed in Straw Yards. Animals (Basel). 2019;9(10):829.

10. Blaxter KL. Experiments with iodinated casein on farms in England and Wales. The Journal of Agricultural Science. 1946;36(2):117-50.

11. Brotherstone S, Coffey MP, Banos G. Genetic Parameters of Growth in Dairy Cattle and Associations Between Growth and Health Traits. Journal of Dairy Science. 2007;90(1):444-50.

12. Brown AD, Pearston F, Mrode R, Kaseja K, Winters M, editors. Lameness evaluations for the UK dairy industry. Interbull Bulletin; 2016 October 24-28; Puerto Varas, Chile2016.

13. Chaplin SJ, Tierney G, Stockwell C, Logue DN, Kelly M. An evaluation of mattresses and mats in two dairy units. Applied Animal Behaviour Science. 2000;66(4):263-72.

14. Clarkson MJ, Downham DY, Faull WB, Hughes JW, Manson FJ, Merritt JB, et al. Incidence and prevalence of lameness in dairy cattle. Veterinary Record. 1996;138(23):563.

15. Collis VJ, Green LE, Blowey RW, Packington AJ, Bonser RHC. Testing White Line Strength in the Dairy Cow. Journal of Dairy Science. 2004;87(9):2874-80.

16. Esslemont RJ, Kossaibati MA. Incidence of production diseases and other health problems in a group of dairy herds in England. Vet Rec. 1996;139(20):486-90.

17. Esslemont RJ, Kossaibati MA. Culling in 50 dairy herds in England. Veterinary Record. 1997;140(2):36.

18. Ferris CP, McCoy MA, Patterson DC, Kilpatrick DJ. Effect of offering dairy cows diets differing in phosphorus concentration over four successive lactations: 2. Health, fertility, bone phosphorus reserves and nutrient utilisation. Animal : an international journal of animal bioscience. 2010;4(4):560-71.

19. Galindo F, Broom D. The relationship between social behaviour of dairy cows and the occurrence of lameness in three herds. Research in veterinary science. 2000;69:75-9.

20. Green LE, Huxley JN, Banks C, Green MJ. Temporal associations between low body condition, lameness and milk yield in a UK dairy herd. Preventive Veterinary Medicine. 2014;113(1):63-71.

21. Griffiths BE, Grove White D, Oikonomou G. A Cross-Sectional Study Into the Prevalence of Dairy Cattle Lameness and Associated Herd-Level Risk Factors in England and Wales. Front Vet Sci. 2018;5:65-.

22. Groenevelt M, Main DCJ, Tisdall D, Knowles TG, Bell NJ. Measuring the response to therapeutic foot trimming in dairy cows with fortnightly lameness scoring. The Veterinary Journal. 2014;201(3):283-8.

23. Haskell MJ, Rennie LJ, Bowell VA, Bell MJ, Lawrence AB. Housing System, Milk Production, and Zero-Grazing Effects on Lameness and Leg Injury in Dairy Cows. Journal of Dairy Science. 2006;89(11):4259-66.

24. Hedges J, Blowey RW, Packington AJ, O’Callaghan CJ, Green LE. A Longitudinal Field Trial of the Effect of Biotin on Lameness in Dairy Cows. Journal of Dairy Science. 2001;84(9):1969-75.

25. Hudson CD, Huxley JN, Green MJ. Using Simulation to Interpret a Discrete Time Survival Model in a Complex Biological System: Fertility and Lameness in Dairy Cows. PLOS ONE. 2014;9(8):e103426.

26. Ivemeyer S, Smolders G, Brinkmann J, Gratzer E, Hansen B, Henriksen BIF, et al. Impact of animal health and welfare planning on medicine use, herd health and production in European organic dairy farms. Livestock production science. 2012;145(1-3):63-72.

27. Kadarmideen HN, Thompson R, Simm G. Linear and threshold model genetic parameters for disease, fertility and milk production in dairy cattle. Animal Science. 2000;71:411-9.

28. Leach KA, Offer JE, Svoboda I, Logue DN. Effects of type of forage fed to dairy heifers: Associations between claw characteristics, clinical lameness, environment and behaviour. The Veterinary Journal. 2005;169(3):427-36.

29. Leach KA, Tisdall DA, Bell NJ, Main DCJ, Green LE. The effects of early treatment for hindlimb lameness in dairy cows on four commercial UK farms. The Veterinary Journal. 2012;193(3):626-32.

30. Lim PY, Huxley J, Willshire JA, Green M, Othman A, Kaler J. Unravelling the temporal association between lameness and body condition score in dairy cattle using a multistate modelling approach. Preventive Veterinary Medicine. 2015;118(4):370-7.

31. Little MW, O’Connell NE, Welsh MD, Barley J, Meade KG, Ferris CP. Prepartum concentrate supplementation of a diet based on medium-quality grass silage: Effects on performance, health, fertility, metabolic function, and immune function of low body condition score cows. Journal of Dairy Science. 2016;99(9):7102-22.

32. Little MW, Arnott GA, Welsh MD, Barley JP, Connell NE, Ferris CP. Comparison of total-mixed-ration and feed-to-yield strategies on blood profiles and dairy cow health. Veterinary Record. 2018;183(21):655.

33. Mahendran SA, Huxley JN, Chang YM, Burnell M, Barrett DC, Whay HR, et al. Randomised controlled trial to evaluate the effect of foot trimming before and after first calving on subsequent lameness episodes and productivity in dairy heifers. The Veterinary Journal. 2017;220:105-10.

34. Manning A. Can negative energy balance in early lactation predict later disease? Cattle Practice. 2018;26(2):79-80.

35. Manson FJ, Leaver JD. The influence of concentrate amount on locomotion and clinical lameness in dairy cattle. Animal Science. 1988;47(2):185-90.

36. March MD, Toma L, Thompson B, Haskell MJ. Food Waste in Primary Production: Milk Loss With Mitigation Potentials. Frontiers in Nutrition. 2019;6(173).

37. Marsman A. The end of OTMS and casualty slaughter: what does it mean to you and your clients? UK Vet: Livestock. 2006;11(3):48...52.

38. Maxwell OJR, Hudson CD, Huxley JN. Effect of early lactation foot trimming in lame and non-lame dairy heifers: a randomised controlled trial. Veterinary Record. 2015;177(4):100.

39. Mill JM, Ward WR. Lameness in dairy cows and farmers' knowledge, training and awareness. Vet Rec. 1994;134(7):162-4.

40. Morris M, Walker S, Jones DN, Routly JE, Smith R, Dobson H. Influence of somatic cell count, body condition and lameness on follicular growth and ovulation in dairy cows. Theriogenology. 2009;71:801-6.

41. Murray RD, Downham DY, Demirkan I, Carter SD. Some relationships between spirochaete infections and digital dermatitis in four UK dairy herds. Research in Veterinary Science. 2002;73(3):223-30.

42. Newsome R, Green MJ, Bell NJ, Chagunda MGG, Mason CS, Rutland CS, et al. Linking bone development on the caudal aspect of the distal phalanx with lameness during life. Journal of Dairy Science. 2016;99(6):4512-25.

43. Newsome RF, Green MJ, Bell NJ, Bollard NJ, Mason CS, Whay HR, et al. A prospective cohort study of digital cushion and corium thickness. Part 1: Associations with body condition, lesion incidence, and proximity to calving. Journal of Dairy Science. 2017;100(6):4745-58.

44. Offer JE, Logue DN, Roberts DJ. The effect of protein source on lameness and solear lesion formation in dairy cattle. Animal Science. 1997;65(2):143-9.

45. Offer JE, McNulty D, Logue DN. Observations of lameness, hoof conformation and development of lesions in dairy cattle over four lactations. Vet Rec. 2000;147(4):105-9.

46. Offer JE, Fisher GEJ, Kempson SA, Logue DN. The Effect of Feeding Grass Silage in Early Pregnancy on Claw Health During First Lactation. The Veterinary Journal. 2001;161(2):186-93.

47. Offer J, Logue D, Offer NW, Marsden M. The effect of concentrate composition on lameness and hoof health in dairy cows. Veterinary journal (London, England : 1997). 2004;167:111-3.

48. Orpin P, Esslemont RJ. Culling and Wastage in Dairy Herds: An Update on Incidence and Economic Impact in Dairy Herds in the UK. Cattle Practice. 2010;18(3):163-72.

49. Phillips CJC. Adverse effects on reproductive performance and lameness of feeding grazing dairy cows partially on silage indoors. The Journal of Agricultural Science. 1990;115(2):253-8.

50. Potterton SL, Green M, Harris J, Millar KM, Whay HR, Huxley J. Risk factors associated with hair loss, ulceration, and swelling at the hock in freestall-housed UK dairy herds. Journal of dairy science. 2011;94:2952-63.

51. Pritchard T, Coffey M, Mrode R, Wall E. Genetic parameters for production, health, fertility and longevity traits in dairy cows. Animal. 2013;7(1):34-46.

52. Pryce JE, Esslemont RJ, Thompson R, Veerkamp RF, Kossaibati MA, Simm G. Estimation of genetic parameters using health, fertility and production data from a management recording system for dairy cattle. Animal Science. 1998;66(3):577-84.

53. Randall LV, Green MJ, Chagunda MGG, Mason C, Archer SC, Green LE, et al. Low body condition predisposes cattle to lameness: An 8-year study of one dairy herd. Journal of Dairy Science. 2015;98(6):3766-77.

54. Randall LV, Green MJ, Chagunda MGG, Mason C, Green LE, Huxley JN. Lameness in dairy heifers; impacts of hoof lesions present around first calving on future lameness, milk yield and culling risk. Preventive Veterinary Medicine. 2016;133:52-63.

55. Randall LV, Thomas HJ, Remnant JG, Bollard NJ, Huxley JN. Lameness prevalence in a random sample of UK dairy herds. Veterinary Record. 2019;184(11):350.

56. Reader J, Green M, Kaler J, Mason S, Green L. Effect of mobility score on milk yield and activity in dairy cattle. Journal of dairy science. 2011;94:5045-52.

57. Rowlands GJ, Lucey S. Changes in milk yield in dairy cows associated with metabolic and reproductive disease and lameness. Preventive Veterinary Medicine. 1986;4(3):205-21.

58. Russell AM, Rowlands GJ, Shaw SR, Weaver AD. Survey of lameness in British dairy cattle. Vet Rec. 1982;111(8):155-60.

59. Rutherford KMD, Langford FM, Jack MC, Sherwood L, Lawrence AB, Haskell MJ. Lameness prevalence and risk factors in organic and non-organic dairy herds in the United Kingdom. The Veterinary Journal. 2009;180(1):95-105.

60. Smith J, van Winden S. Risk of Lameness in Dairy Cows with Paratuberculosis Infection. Animals (Basel). 2019;9(6):339.

61. Thomas HJ, Remnant JG, Bollard NJ, Burrows A, Whay HR, Bell NJ, et al. Recovery of chronically lame dairy cows following treatment for claw horn lesions: a randomised controlled trial. Veterinary Record. 2016;178(5):116.

62. Walker SL, Smith RF, Jones DN, Routly JE, Dobson H. Chronic stress, hormone profiles and estrus intensity in dairy cattle. Hormones and Behavior. 2008;53(3):493-501.

63. Walker S, Smith R, Routly JE, Jones DN, Morris M, Dobson H. Lameness, Activity Time-Budgets, and Estrus Expression in Dairy Cattle. Journal of dairy science. 2009;91:4552-9.

64. Weaver AD. Claw trimming: what farmers think! Cattle Practice. 1997;5:23-5.

65. Weller RF, Cooper A. Health status of dairy herds converting from conventional to organic dairy farming. The Veterinary record. 1996;139(6):141-2.

66. Whitaker DA, Kelly JM, Smith EJ. Incidence of lameness in dairy cows. The Veterinary record. 1983;113(3):60-2.

67. Whitaker DA, Kelly JM, Smith S. Disposal and Disease Rates in 340 British Dairy Herds. The Veterinary record. 2000;146:363-7.

68. Whitaker DA, Macrae AI, Burrough E. Disposal and disease rates in British dairy herds between April 1998 and March 2002. Veterinary Record. 2004;155(2):43.

69. White A. Long standing lameness problem in a dairy herd. UK Vet: Livestock. 2011;16(2):29-33.

70. Ivemeyer S, Smolders G, Brinkmann J, Gratzer E, Hansen B, Henriksen BIF, et al. Impact of animal health and welfare planning on medicine use, herd health and production in European organic dairy farms. Livestock Science. 2012;145(1):63-72.

71. Bell N. Case report: Reducing lameness through active screening, attention to details and skilled staff. Livestock. 2012;17.

72. Little M, Arnott G, Welsh M, Barley J, Connell N, Ferris C. Comparison of total-mixed-ration and feed-to-yield strategies on blood profiles and dairy cow health. Veterinary Record. 2018;183:vetrec-2017.
