# Supplementary figures and images for: Profiling Detection and Classification of Lameness Methods in British Dairy Cattle Research: A Systematic Review and Meta-Analysis
Source: Front Vet Sci. 2020 Aug 20;7:542. doi: 10.3389/fvets.2020.00542 (PMC7468474; doi:10.3389/fvets.2020.00542)

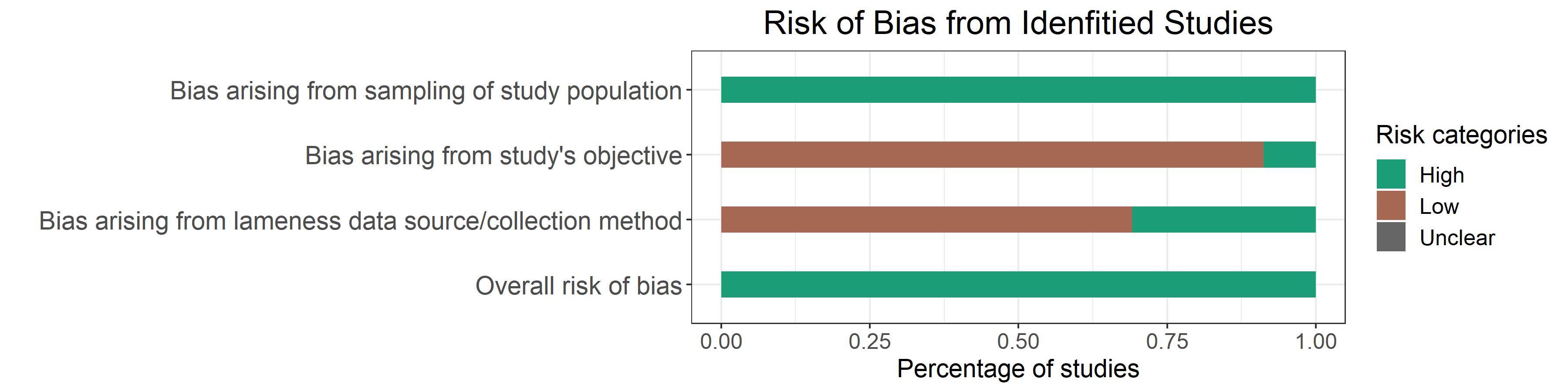

Supplement: Supplementary file 2 [file Image_1.JPEG]
